# Integrative View of the Diversity and Evolution of SWEET and SemiSWEET Sugar Transporters

**DOI:** 10.3389/fpls.2017.02178

**Published:** 2017-12-20

**Authors:** Baolei Jia, Xiao Feng Zhu, Zhong Ji Pu, Yu Xi Duan, Lu Jiang Hao, Jie Zhang, Li-Qing Chen, Che Ok Jeon, Yuan Hu Xuan

**Affiliations:** ^1^School of Bioengineering, Qilu University of Technology, Jinan, China; ^2^Department of Life Sciences, Chung-Ang University, Seoul, South Korea; ^3^College of Plant Protection, Shenyang Agricultural University, Shenyang, China; ^4^School of Life Science and Biotechnology, Dalian University of Technology, Dalian, China; ^5^Department of Plant Biology, University of Illinois at Urbana–Champaign, Urbana, IL, United States

**Keywords:** SWEET, SemiSWEET, evolution, sequence similarity network, gene fusion, biosphere

## Abstract

Sugars Will Eventually be Exported Transporter (SWEET) and SemiSWEET are recently characterized families of sugar transporters in eukaryotes and prokaryotes, respectively. SemiSWEETs contain 3 transmembrane helices (TMHs), while SWEETs contain 7. Here, we performed sequence-based comprehensive analyses for SWEETs and SemiSWEETs across the biosphere. In total, 3,249 proteins were identified and ≈60% proteins were found in green plants and Oomycota, which include a number of important plant pathogens. Protein sequence similarity networks indicate that proteins from different organisms are significantly clustered. Of note, SemiSWEETs with 3 or 4 TMHs that may fuse to SWEET were identified in plant genomes. 7-TMH SWEETs were found in bacteria, implying that SemiSWEET can be fused directly in prokaryote. 15-TMH extraSWEET and 25-TMH superSWEET were also observed in wild rice and oomycetes, respectively. The transporters can be classified into 4, 2, 2, and 2 clades in plants, Metazoa, unicellular eukaryotes, and prokaryotes, respectively. The consensus and coevolution of amino acids in SWEETs were identified by multiple sequence alignments. The functions of the highly conserved residues were analyzed by molecular dynamics analysis. The 19 most highly conserved residues in the SWEETs were further confirmed by point mutagenesis using SWEET1 from *Arabidopsis thaliana*. The results proved that the conserved residues located in the extrafacial gate (Y57, G58, G131, and P191), the substrate binding pocket (N73, N192, and W176), and the intrafacial gate (P43, Y83, F87, P145, M161, P162, and Q202) play important roles for substrate recognition and transport processes. Taken together, our analyses provide a foundation for understanding the diversity, classification, and evolution of SWEETs and SemiSWEETs using large-scale sequence analysis and further show that gene duplication and gene fusion are important factors driving the evolution of SWEETs.

## Introduction

Sugars are ubiquitous compounds that play important roles in living organisms, including involvement in osmotic regulation, cell signaling, energy storage, and carbon skeletons (Chen et al., 2015). One major function of sugars is their use for energy within living organisms. Glucose is a commonly known carbohydrate that is metabolized in cells to create fuel and that is the energy source for brain tissue, neurons, and developing red blood cells. Sugars are precursors for the biosynthesis of many other cellular compounds and serve as critical signaling molecules related to both cellular metabolic status and stress responses (Ramon et al., 2008; Mergenthaler et al., 2013).

Sugar metabolism has been extensively studied, and the major sugar metabolic pathways are well established. A first step before the catabolism of an exogenous sugar is usually transport across a cell membrane, and the exchange of sugars between different cells in organisms makes sugar transport a critical process. Organisms have evolved several mechanisms to transport sugars across membranes, and the understanding of carbohydrate transport processes that are facilitated by sugar transporters is becoming increasingly sophisticated (Lalonde et al., 2004; Chen et al., 2015). These transporters can be classified into mainly three superfamilies: major facilitator superfamily (MFS) transporters, sodium-solute symporter family (SSF) transporters, and the newly identified Sugars Will Eventually be Exported Transporters (SWEETs) and SemiSWEETs families (Chen et al., 2015). MFS transporters are conserved from bacteria to humans and include the bacterial lac permease, yeast hexose transporters (HXTs), human glucose transporters (GLUTs), and plant sugar transporters (Quistgaard et al., 2016). SSF proteins catalyze sugar uptake across the cytoplasmic membranes of pro- and eukaryotic cells via the electrochemical sodium gradient (sodium motive force) (Jung, 2002). SWEETs catalyze the facilitated diffusion of sugars driven by their concentration gradients (Deng and Yan, 2016). The SWEETs in eukaryotes typically consist of seven transmembrane helices (TMHs), including a pair of 3-TMH repeats and an additional helix connecting these two repeats. In contrast, the bacterial SemiSWEETs possess 3 TMHs in total (Xuan et al., 2013).

SWEETs and SemiSWEETs are present in all kingdoms of life. In plants, approximately 20 SWEET paralogs, which may transport mono- and/or disaccharides, can be identified in most genomes (Feng and Frommer, 2016). The SWEETs in plants participate in important physiological processes, including phloem transport, nectar secretion, pollen nutrition, stress tolerance, and plant-microbe interactions (Yang et al., 2006; Chen et al., 2012; Chardon et al., 2013; Lin et al., 2014). The human genome contains only one *SWEET* gene, which has a broad expression pattern and which mediates glucose transport (Chen et al., 2010). The Swt-1 in *Caenorhabditis elegans* mediates glucose and trehalose transport, and suppression of its expression by RNAi is reported to be associated with lipid accumulation (Palgunow, 2012). BjSemiSWEET (from *Bradyrhizobium japonicum*) transports sucrose, but its physiological function is not clear (Xuan et al., 2013).

The crystal structures of OsSWEET2b from rice (*Oryza sativa*) (Tao et al., 2015) and SemiSWEETs from *Vibrio* sp. N418, *Leptospira biflexa, Thermodesulfovibrio yellowstonii*, and *Escherichia coli* have been determined (Wang et al., 2014; Xu et al., 2014; Lee et al., 2015). OsSWEET2b forms a homotrimeric complex in which the fourth TMH interacts closely with the first 3-TMH repeats and mediates key contacts among the protomers (Tao et al., 2015). SemiSWEETs form dimeric complexes, and each protomer undergoes an intramolecular conformational change during sugar transport (Lee et al., 2015; Latorraca et al., 2017).

Phylogenetic studies have revealed that SWEETs and SemiSWEETs are related to the PQ-loop family, which shares the same TMH architecture and possesses a signature motif with Pro-Gln residues (PQ-loop motif) in the first TMH (Eom et al., 2015; Feng and Frommer, 2015). From an evolutionary perspective, eukaryotic SWEETs may have originated from fusion between an archaeal and a bacterial SemiSWEET (Feng and Frommer, 2015; Hu et al., 2016), but further information is needed to dissect the evolutionary route from SemiSWEETs to SWEETs. To gain insights into the evolution and diversity of SWEETs and SemiSWEETs across the biosphere, we performed a comprehensive study using sequence similarity networks (SSNs), phylogenetic trees, sequence alignments and experimental analyses. We found that the SWEET sequences were more highly variable than had previously been estimated and gene duplication/fusion played important roles during evolution.

## Materials and methods

### Collection of functional and putative SWEETs and SemiSWEETs

For a global analysis of SWEETs and SemiSWEETs, the sequences of these genes were retrieved from the InterPro database (http://www.ebi.ac.uk/interpro/entry/IPR004316; release 59.0) (Mitchell et al., 2015). Redundant sequences were removed by CD-HIT, with a requirement of 100% identity (Li and Godzik, 2006). The fragment sequences were removed. The proteins were screened for the presence of the PQ-loop motif using the NCBI conserved domain database and Pfam (Marchler-Bauer et al., 2015) and for the presence of at least three TMHs using TMHMM Server v.2.0 (Krogh et al., 2001). The proteins identified in the database are listed in Supplemental Dataset.

### Construction of SSNs

SSNs based on the retrieved proteins were constructed using the Enzyme Function Initiative-Enzyme Similarity Tool (EFI-EST) (Gerlt et al., 2015) and visualized using Cytoscape 3.3 (Shannon et al., 2003). Each node in the network represents a protein, and an edge indicates that the two nodes connected by that edge are significantly similar, having an *e*-value less than the selected cutoff (Jia et al., 2017a,b,c).

### Multiple sequence alignments (MSAs) and coevolving protein residues

MSAs of protein sequences were carried out using the ClustalW (version 2) program (Larkin et al., 2007), and graphics of the conserved residues were generated using Seq2Logo (Thomsen and Nielsen, 2012). The unrooted phylogenetic trees were constructed with MEGA7 using maximum likelihood (ML) methods and bootstrapping with 1,000 iterations (Jia et al., 2015; Kumar et al., 2016). Analysis of coevolving residues was performed using mutual information (MI) between two positions in the MSAs, which indicates the extent to which knowledge of the amino acid at one position can allow prediction of the amino acid at the other position. MI was determined between pairs of columns in the MSA using the MISTIC web server (Simonetti et al., 2013).

### Molecular dynamics (MD) simulation and binding free energy calculation

The starting structure used for MD simulation was downloaded from the Protein Data Bank (PDB ID: 5CTG), and missing residues were filled in using GalaxyFill (Coutsias et al., 2004). The ligand-bound form was created using the docking of SWEET and glucose in the LeDock program (Hartshorn et al., 2007). SWEET-glucose complexes were embedded in a preequilibrated lipid bilayer for membrane dynamics simulation studies (Schmidt and Kandt, 2012). The lipid bilayer system for these simulations was constructed from a palmitoyloleoylphosphatidylcholine (POPC) lipid bilayer, which is the major constituent of eukaryotic cell membranes. The initial lipid bilayer consisted of 300 lipid molecules but was reduced to a system with 228 lipid molecules (Pluhackova et al., 2016). POPC lipids used a Lipid14 force field model. Without position restraints to satisfy the bilayer parameters such as area per lipid, order parameters for the palmitoyl and oleoyl chains and electron density profiles were obtained with the experimental results. The GROMACS topology for glucose was obtained from ACPYPE (Sousa da Silva and Vranken, 2012). The resulting system was solvated, followed by the addition of ions (0.15 M NaCl) to neutralize the system. The total system was energy minimized, and 400-ps equilibration was carried out on the solvent and ions with position restraints on the rest of the system. The equilibration process was proceeded for an additional 10 ns with position restraints on the protein to allow the appropriate positioning of lipid molecules around the protein. Two separate simulations were carried out on the apo- and glucose-bound forms of SWEET. All these simulations were performed using the GROMACS 5.0.4 program with the Amber ff14SB force field and a constant temperature and pressure ensemble. Nose-Hoover coupling was used to keep a constant temperature of 300 K and constant semiisotropic pressure of 1 bar with coupling times of 0.5 and 5 ps. Separate temperature coupling of protein, POPC and ligand, solvent and ions was carried out with a coupling constant of 0.5 ps. The distance cut-off for the Coulomb and Lennard-Jones interactions was 1.2 nm. The particle-mesh Ewald method was employed to treat long-range electrostatic interactions. All bonds were constrained with the LINCS algorithm (Hess et al., 1997). The time step used was 2 fs, and the coordinates were saved every 10 ps for analysis. The gmx rmsd and gmx rmsf programs in the GROMACS 5.0.4 was used to obtain the root-mean-square deviation (RMSD) and root-mean-square fluctuation (RMSF), respectively (Kumari et al., 2014; Genheden and Ryde, 2015).

Two hundred fifty snapshots were retrieved from the last 25 ns along the MD trajectory at an interval of 100 ps. The molecular mechanic/Poisson-Boltzmann surface area (MM/PBSA) method was employed using the g_mmpbsa package (Kumari et al., 2014; Genheden and Ryde, 2015) to calculate the binding free energies of the protein and substrate. The MM/PBSA method can be conceptually summarized as three energetic terms (Wang et al., 2016):

(1) $$\begin{array}{r} \Delta G_{total}=\Delta E_{MM}+\Delta G_{sol}-T\Delta S \end{array}$$

where Δ*G*_*total*_ represents the binding free energy, Δ*E*_*MM*_ denotes the difference in molecular mechanics energy between the complex and each binding partner in a vacuum, Δ*G*_*sol*_ represents the solvation free energy, and TΔ*S* represents the entropy change. Δ*E*_*MM*_ can be further divided into the two following parts:

(2) $$\begin{array}{r} \Delta E_{MM}=\Delta E_{ele}+\Delta E_{vdw} \end{array}$$

where Δ*E*_*ele*_ and Δ*E*_*vdw*_ represent the electrostatic interaction, and van der Waals energy in a vacuum, respectively. In addition, the solvation free energy can be divided into two parts:

(3) $$\begin{array}{r} \Delta G_{sol}=\Delta G_{polar}+\Delta G_{np} \end{array}$$

where Δ*G*_*polar*_ and Δ*G*_*np*_ represent the polar and non-polar solvation free energies, respectively. For Δ*G*_*polar*_, the default values for the dielectric constants of the solute and solvent were used in our calculations. For Δ*G*_*np*_, the values of coefficients γ and β were set to 0.0054 kcal/mol/A^2^ and 0.92 kcal/mol, respectively.

The entropy change (TΔ*S*) arises from changes in the translational, rotational, and vibrational degrees of freedom. The calculation of entropy change is extremely time-consuming and inaccurate, and for similar SWEET-glucose complex systems, the entropy changes are similar (Homeyer and Gohlke, 2012). Therefore, we ignored the calculation of entropy change in our study.

### Vector construction

To clone wild-type *AtSWEET1* and prepare mutant *AtSWEET1* constructs with changes to conserved residues, PCR-based mutagenesis was performed. The primer sequences used for cloning are listed in Supplemental Table 1. PCR fragments were purified and cloned into the Gateway entry vector pDONR221-f1 and subsequently sequenced by Sangon Biotech (Shanghai, China) (Jia and Jeon, 2016). Correct entry clone plasmids were mixed with the destination vector pDRf1 GW for the construction of yeast expression vectors via LR reactions (Xuan et al., 2013).

### Functional analysis of AtSWEET1 mutants in yeast

The yeast hexose transporter mutant strain EBY4000 [hxt1-17D::loxPgal2D::loxP stl1D::loxP agt1D::loxP ydl247wD::loxP yjr160cD::loxP] was used to test hexose transport activity (Wieczorke et al., 1999). Wild-type and mutant AtSWEET1 were expressed in EBY4000 using the pDRf1-GW vector. Transformants were selected on solid SD (-ura) medium with 2% maltose as the carbon source at 28°C for 3 days. Subsequently, the growth of the yeast cells on SD medium containing 2% glucose or maltose was monitored. Plates were incubated at 28°C for 3 days.

## Results

### Taxonomic distribution and classification of SWEET and SemiSWEET homologs

**Figure 1:**
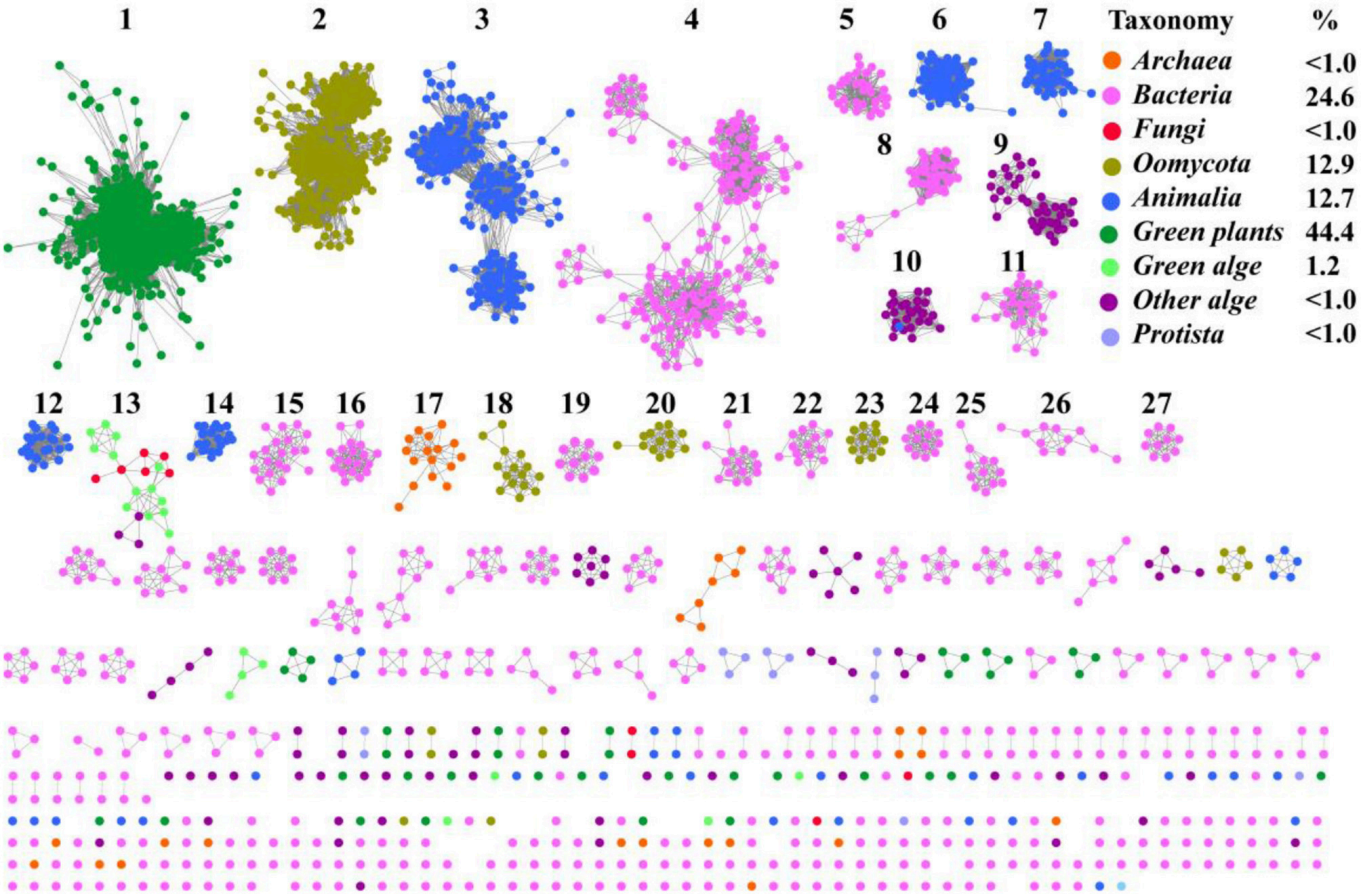
Taxonomic distribution of SWEETs and SemiSWEETs. The proteins listed in S1 Dataset were used to generate the network, using an e-value of 10^−55^ (>50% sequence identity). Each node represents one protein. Edges are shown with e-values below the indicated cutoff. Clusters containing more than 10 nodes are labeled sequentially. Nodes from the same taxonomic groups in the global network are the same color. The colors corresponding to different taxonomic groups are listed on the right. Taxonomic classifications included were the class Oomycota; the phyla *Streptophyta* (green plants), *Chlorophyta* (green algae), and other algae; the kingdoms, Metazoa, Fungi, and Protista; and the superkingdoms Bacteria and Archaea. The colors corresponding to the different taxonomic groups and the percentage of proteins in each group are listed on the right.

To understand the distribution of SWEETs and SemiSWEETs in the biosphere, protein sequences were acquired from the InterPro database (InterPro family IPR004316; release 59.0). Proteins containing a P-Q loop motif and more than three TMHs were selected for further analysis. In total, 3,249 proteins were obtained (Supplemental Dataset). To further clarify the distribution of and relationships among these proteins, a network for the 3,249 protein sequences was constructed with an *e*-value threshold of 10^−55^. Each network shown had the same *e*-value (>50% sequence identity was required to draw an edge between nodes), and the proteins were grouped into 27 clusters that each contained 10 or more members. Each protein was painted according to taxonomic classification (Figure 1). Proteins are found in superkingdoms Archaea and Bacteria; kingdoms Metazoa, Fungi, and Protista; phyla of *Streptophyta* (green plants), *Chlorophyta* (green algae), and other algae; and class Oomycota. Of these proteins, 44.4% are found in green plants. The numbers of proteins in Metazoa and Oomycota are similar, accounting for 12.7 and 12.9%, respectively. Bacteria contain the fourth highest percentage (24.6%). SWEETs from algae, including *Bacillariophyta, Chlorophyta, Cryptophyta, Haptophyta*, and *Rhodophyta*, account for 1.2% of all SWEETs examined. Previously, 3 SWEETs and 1 putative SemiSWEET were found in Fungi, while 2 SemiSWEETs were observed in Archaea (Hu et al., 2016). In our study, 41 and 11 proteins were discovered in Archaea and Fungi, respectively. Other SWEET proteins are found in Protista such as Alveolata, Amoebozoa, and Euglenozoa.

Most of the SWEET proteins from plants cluster together (cluster 1), indicating that they may have arisen from the same ancestor. Some proteins from oomycetes form the second largest cluster (cluster 2), and others can be found in clusters 18, 20, and 23. Proteins from Metazoa show greater diversity and can be separated into 5 clusters (clusters 3, 6, 7, 12, and 14). SemiSWEETs from Archaea are located in cluster 17. Compared with the SWEETs and SemiSWEETs from Archaea and eukaryotes, the bacterial proteins demonstrate the highest diversity and are classified into more clusters. In addition, all the clusters except 13, which includes proteins from both fungi and algae, contain proteins belonging to the same taxonomic classification. This suggests that the evolution and distribution of SWEETs and SemiSWEETs have been very well-conserved.

**Figure 2:**
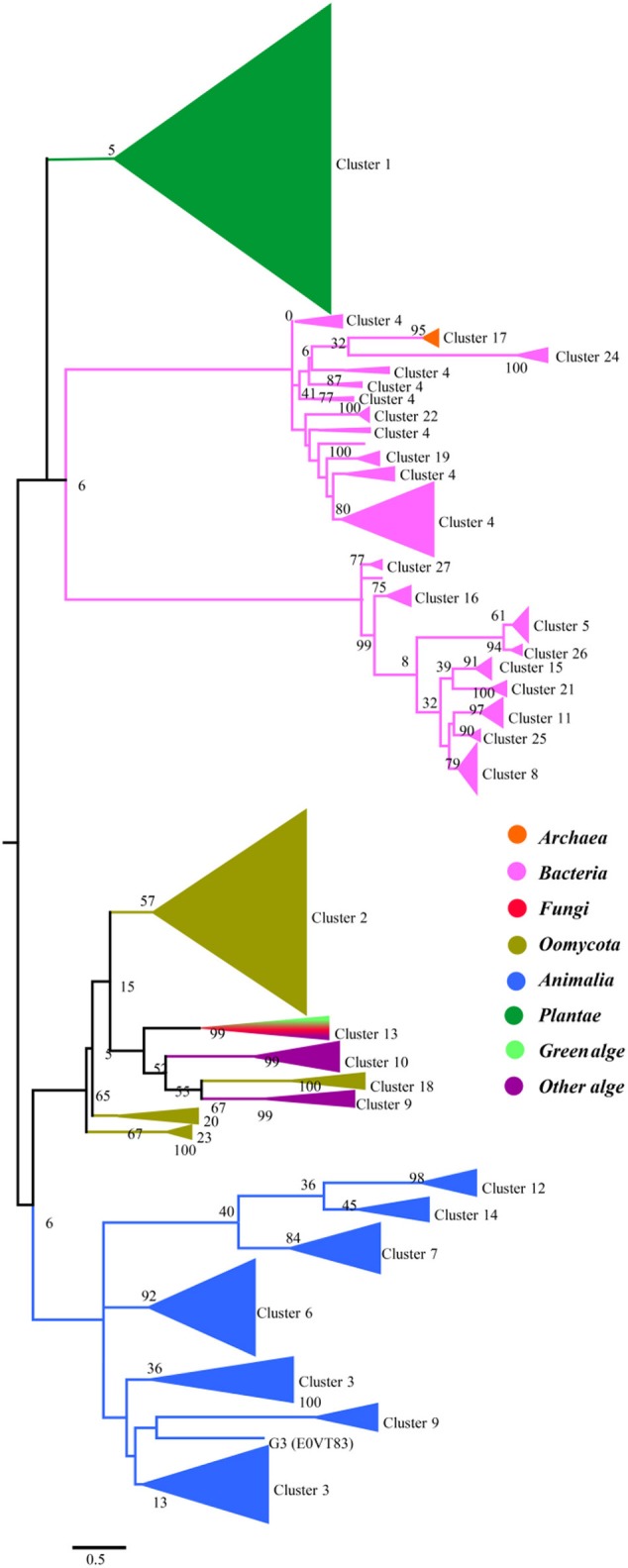
Maximum likelihood phylogenetic tree for SWEETs and SemiSWEETs generated using MEGA. The tree with the highest log likelihood (−325820.9727) is shown. The percentage of replicate trees in which the associated taxa clustered together in the bootstrap test (1,000 replicates) is shown next to the branches. The color of each branch corresponds to the color of its cluster in Figure 1.

To gain a detailed view of the evolutionary relationships across the groups, we performed a phylogenetic analysis using the proteins in the clusters assigned based on sequence comparisons (Figure 2). Proteins from the same cluster always grouped together and, with only a few exceptions, are well separated in the phylogenetic tree. The separation of these clusters has high levels of bootstrap support in the phylogenetic tree. These proteins can be further classified into four groups: plants, prokaryotes, oomycetes/algae, and Metazoa, although the bootstrap values are low because of the large amount of sequences in the analysis. In the protein SSNs, the SWEETs from plants form a single and the largest cluster (Cluster 1). These proteins also form a separate branch in the phylogenetic tree. The proteins in Cluster 17, from Archaea, are gathered in a clade with a high level of bootstrap support, and these proteins are most similar to the proteins from bacteria (Cluster 24). The bacterial proteins form multiple branches that correspond to the clusters shown in Figure 1, which suggests that the prokaryotic SemiSWEETs are much more diverse in sequence than the eukaryotic SWEETs. SWEETs from oomycetes are closest to the proteins from algae in the phylogenetic tree. Cluster 13, containing SWEETs from fungi and algae, is located between the oomycetes and algae. The proteins from metazoa form the last group, including Clusters 3, 6, 9, 12, and 14. In conclusion, SemiSWEETs and SWEETs can be classified into four groups: plants, prokaryotes, oomycetes/algae, and metazoa, based on the degrees of relatedness among these proteins and their phylogenetic neighbors.

### Diversity of TMHs in SWEETs and SemiSWEETs

**Figure 3:**
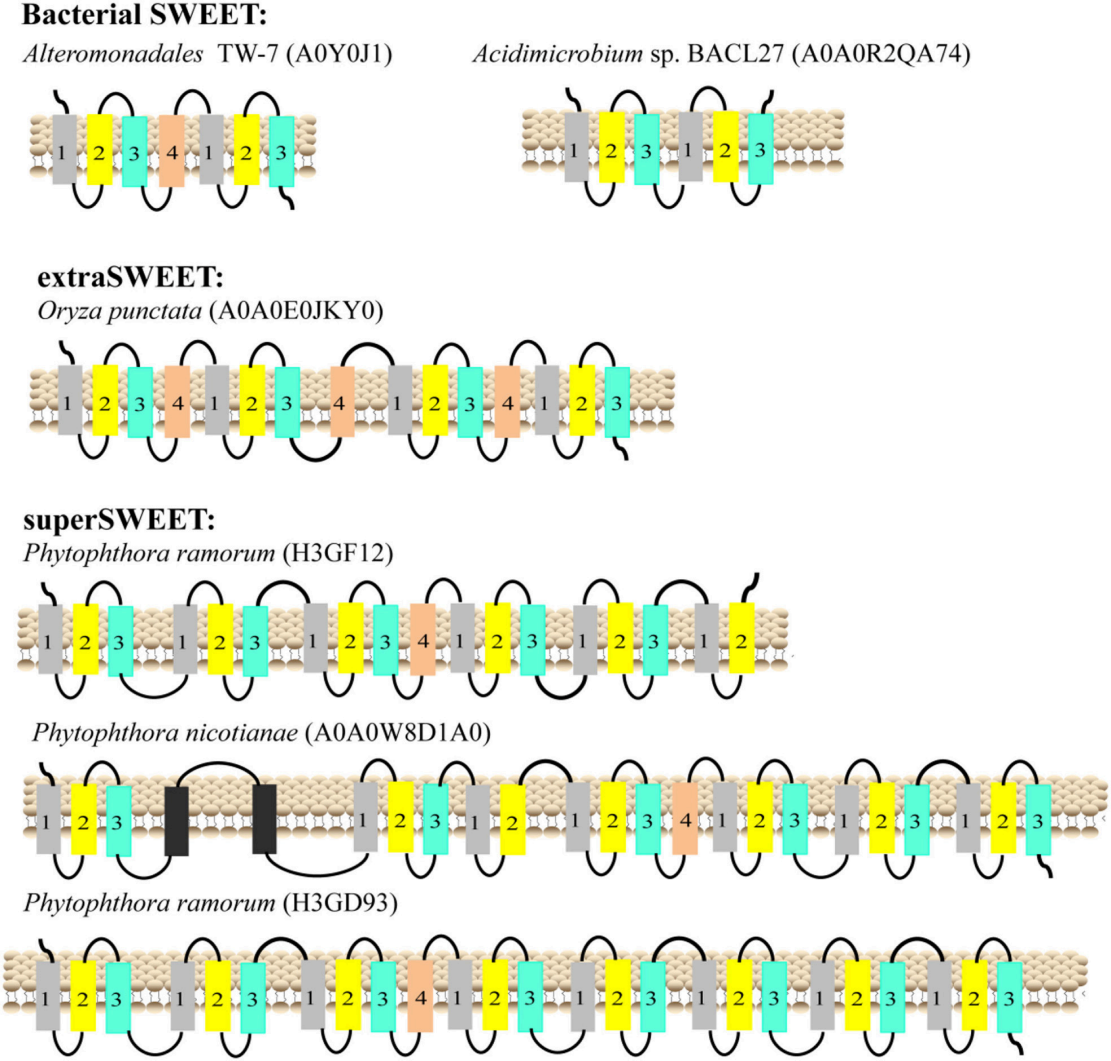
Schematic two-dimensional model of SWEETs with multi-units characteristics from bacteria, plants, and oomycetes. TMH1, TMH2, TMH3, and TMH4 are shown in gray, yellow, blue and orange, respectively. The unknown transmembrane helix is shown in black. The origins of the proteins and their UniProt IDs are shown above the corresponding models.

The typical SemiSWEETs in prokaryotes contain 3 TMHs to mediate sugar transport, and the typical SWEET proteins in eukaryotes consist of two tandem repeats of 3 TMHs separated by a single TMH, which evolved by internal duplication of the 3 TMHs (Feng and Frommer, 2015). However, both 7-TMH SWEETs and 3-TMH SemiSWEETs were identified in eukaryotes (Supplemental Dataset and Supplemental Table 2). Interestingly, an extraSWEET protein from *Vitis vinifera*, consisting of 14 TMHs, has recently been reported (Patil et al., 2015). This novel extraSWEET might be the result of an internal duplication of 7 TMHs, similar to the duplication of SemiSWEET that produced SWEET. To better understand the diversity of the TMHs, their topology was estimated using TMHMM (Supplemental Table 2). The results show that all the proteins from archaea are SemiSWEETs. Most of the bacterial homologs are SemiSWEETs, but some bacterial homologs with 6 or 7 TMHs are also found (Figure 3). In eukaryotes, proteins with 6 or 7 TMHs are dominant, but SemiSWEETs with 3 or 4 TMHs are found in fungi, plants, metazoa, oomycetes, algae, and protists. The extraSWEET from *V. vinifera* was also identified (UniProt ID F6I4N9) (Supplemental Dataset and Supplemental Table 2). One protein from *Oryza punctata* (A0A0E0JKY0) contains 15 TMHs (Figure 3). Interestingly, three superSWEETs from oomycetes (H3GF12, A0A0 W8D1A0, and H3GD93) contain 18, 23, and 25 TMHs (Figure 3), respectively, which represent more than five duplications of SemiSWEET. The increase in the number of TMHs in SWEETs indicates that duplication and fusion may be an important process for the evolution of these proteins.

### Phylogenetic analysis of SWEETs in green plants

**Figure 4:**
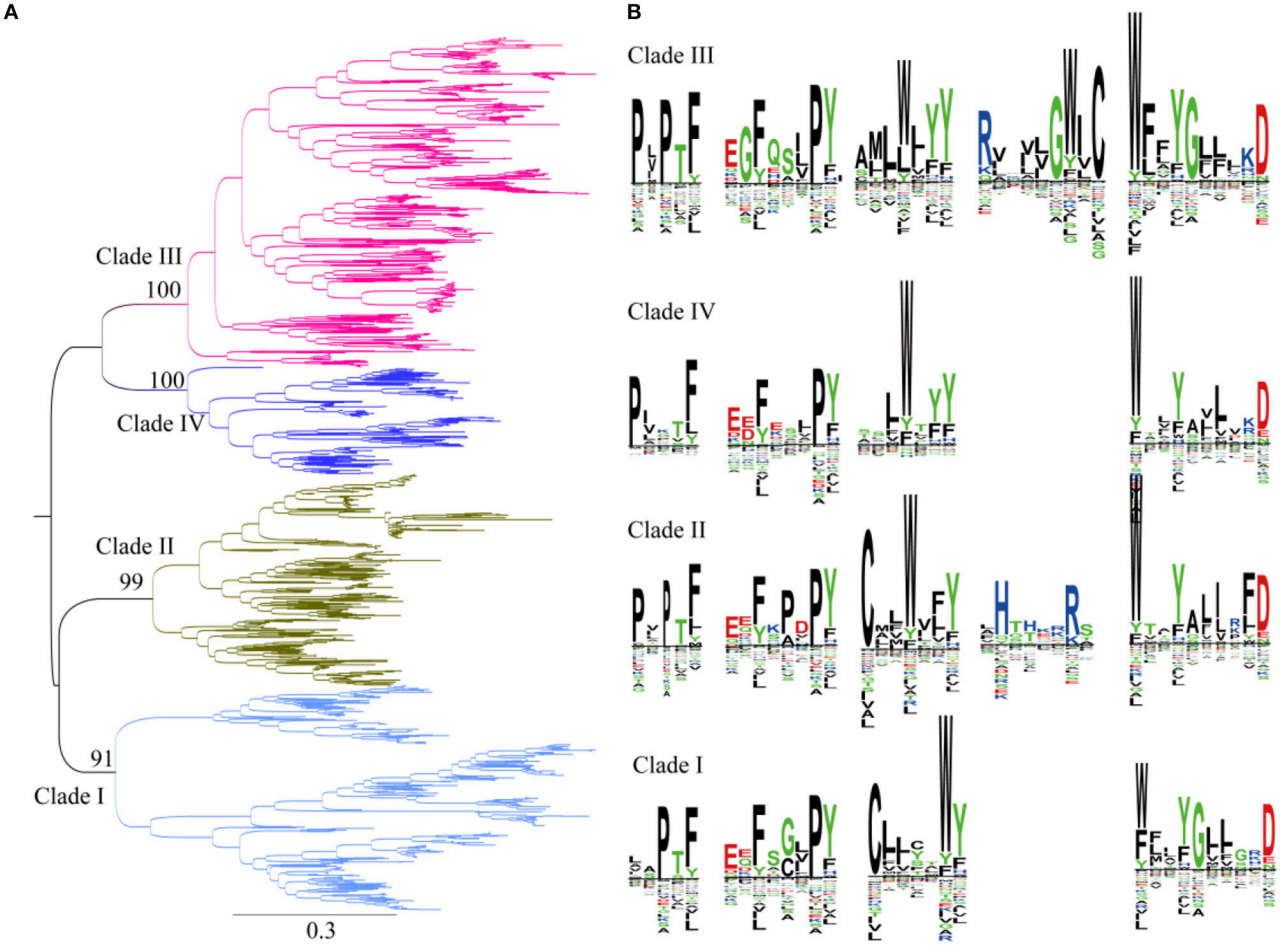
Phylogenetic and conserved motif analyses of SWEETs from green plants. **(A)** Molecular phylogenetic analysis by the maximum likelihood method. The tree with the highest log likelihood (-261256.9227) is shown. The tree is drawn to scale, with branch lengths indicating the number of substitutions per site. There was a total of 2,335 positions in the final dataset. Evolutionary analyses were conducted in MEGA7. **(B)** Sequence alignment of conserved motifs of SWEETs in different clades corresponding to **(A)**. The sequences were aligned by ClustalW. The conservation level of each residue is indicated by the height of the bar above it. The sequence logo was generated using Seq2Logo.

Previous studies showed that plant SWEETs fall into four clades (Chandran, 2015; Eom et al., 2015). Members of SWEETs in one clade does not seem to indicate that they carry out the same physiological process but does appear to be in accordance with relative selectivity for mono- vs. disaccharides (Eom et al., 2015). SWEETs belonging to clades I, II, and IV preferentially transport glucose, galactose, and/or fructose, whereas clade III members function primarily as disaccharide transporters. In addition, clade IV SWEETs localized to the vacuolar membrane (Chandran, 2015). To explore the evolutionary relationships among plant SWEETs, a phylogenetic tree was constructed using the amino acid sequences of plant SWEETs covering 59 species (Figure 4A). Interestingly, the high bootstrap values indicated that all the plant proteins from the database also cluster into four clades. Clade I is most close to clade II, while clades III and IV are in the same branch. To further illustrate the differences in sequences, the proteins in each clade were aligned and the conserved motifs displayed using the SWEETs from *Arabidopsis* as reference sequences (AtSWEET1 for clade I, AtSWEET4 for clade II, AtSWEET9 for clade III, and AtSWEET16 for clade IV). Four or five conserved motifs can be identified in each clade. In all the clades, two conserved motifs in the N-terminus and one in the C-terminus are almost identical (Figure 4B). The main difference is found in the central part of the protein: both cysteine and tryptophan residues are highly conserved in TMH2 in clades I and II, but only tryptophan is conserved in clades III and IV. The comparison of clade I with clade II shows that clade II has an additional conserved motif with two positively charged amino acids (Arg and His). Comparison of clade III with clade IV shows that clade III contains an additional motif with highly conserved Arg and Trp residues.

### Phylogenetic analysis of SWEETs in metazoa

**Figure 5:**
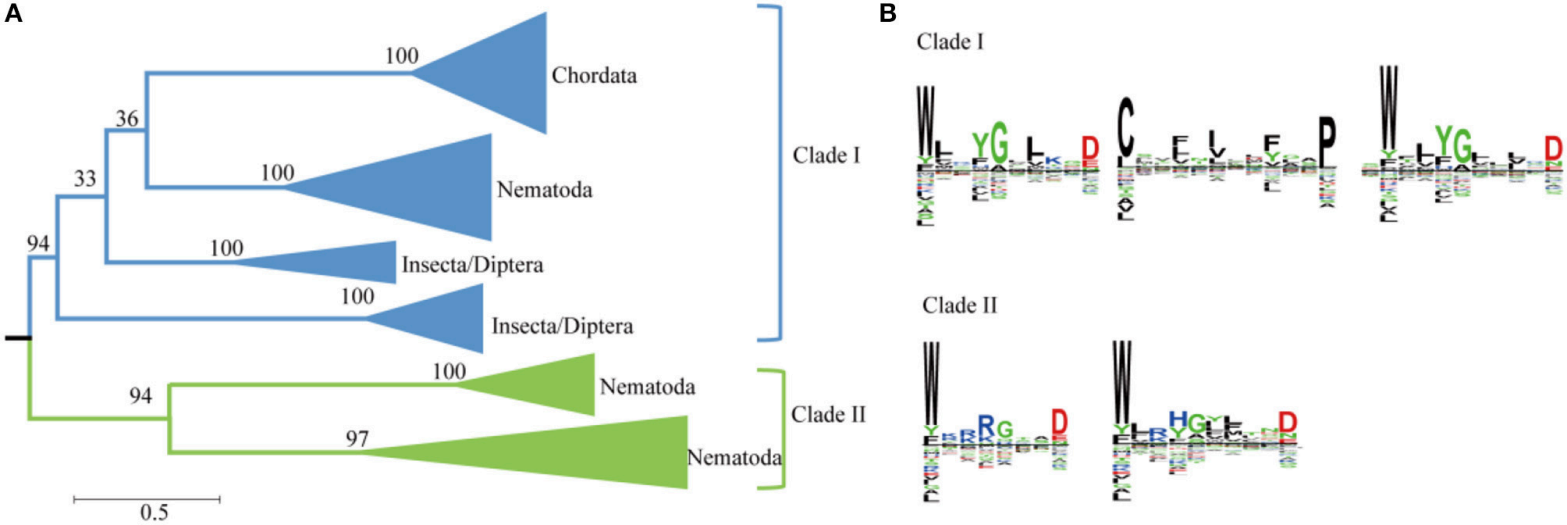
Phylogenetic and conserved motif analyses of SWEETs from Metazoa. **(A)** Molecular phylogenetic analysis by the maximum likelihood method. The tree with the highest log likelihood (−35684.4811) is shown. The tree is drawn to scale, with branch lengths indicating the number of substitutions per site. Evolutionary analyses were conducted in MEGA7. **(B)** Sequence alignment of conserved motifs of SWEETs in different clades corresponding to **(A)**. The sequences were aligned by ClustalW. The conservation level of each residue is indicated by the height of the bar above it. The sequence logo was generated using Seq2Logo.

Plant genomes typically contain more than 10 *SWEETS*; however, the human and *Drosophila* genomes each contain only one SWEET (Chen et al., 2010; Yuan and Wang, 2013). A phylogenetic analysis using Metazoa SWEET proteins indicates that they are divided into six clusters and two major clades (Figure 5A). The first clade includes the classical human SWEET, which is clustered with the proteins from Teleostomi in the phylum Chordata, which includes fish (Zebrafish), amphibians (African clawed frog), reptiles (green sea turtle), birds (collared flycatcher), and Mammalia (house mouse). These organisms function with one copy of SWEET (Supplemental Figure 1). This cluster, together with the proteins from Insecta and Nematoda, forms the first clades. Within Insecta, SWEETs are found in Diptera (*Drosophila, Anopheles*, and others). In *Drosophila melanogaster*, a favorite model organism for genetics, two SWEETs exist (Q9VUN8 and A0A0B4LET7, located on chromosomes 3R and 2L, respectively). The same variables are also present in other Diptera, causing the proteins from Diptera to form two clusters. Moreover, members of Nematoda possess at least one SWEETs. Seven SWEETs (O16448, Q21254, Q17757, O45102, O44620, and P92011, and Q9XX26) are present in *Caenorhabditis elegans*, a model organism for the investigation of neural development in Metazoa. These proteins are separated into three clusters. The proteins in the first cluster are with the orthologs from insects and mammals. Meanwhile, the paralogs from the other two clusters of Nematoda form another clade with a high level of bootstrap support. The separation of these clusters has a high level of bootstrap support in the phylogenetic tree. In both clades, the protein sequences contain two highly conserved WX_n_D motifs (Figure 5B). In clade I, one motif is located in the N-terminus and the other in the C-terminus, while both motifs are located in N-terminus in clade II. In clade I, a motif beginning with Cys and ending with Pro, between the two WX_n_D motifs, is also conserved.

### Phylogenetic analysis of SWEETs in unicellular eukaryotes and fungi

**Figure 6:**
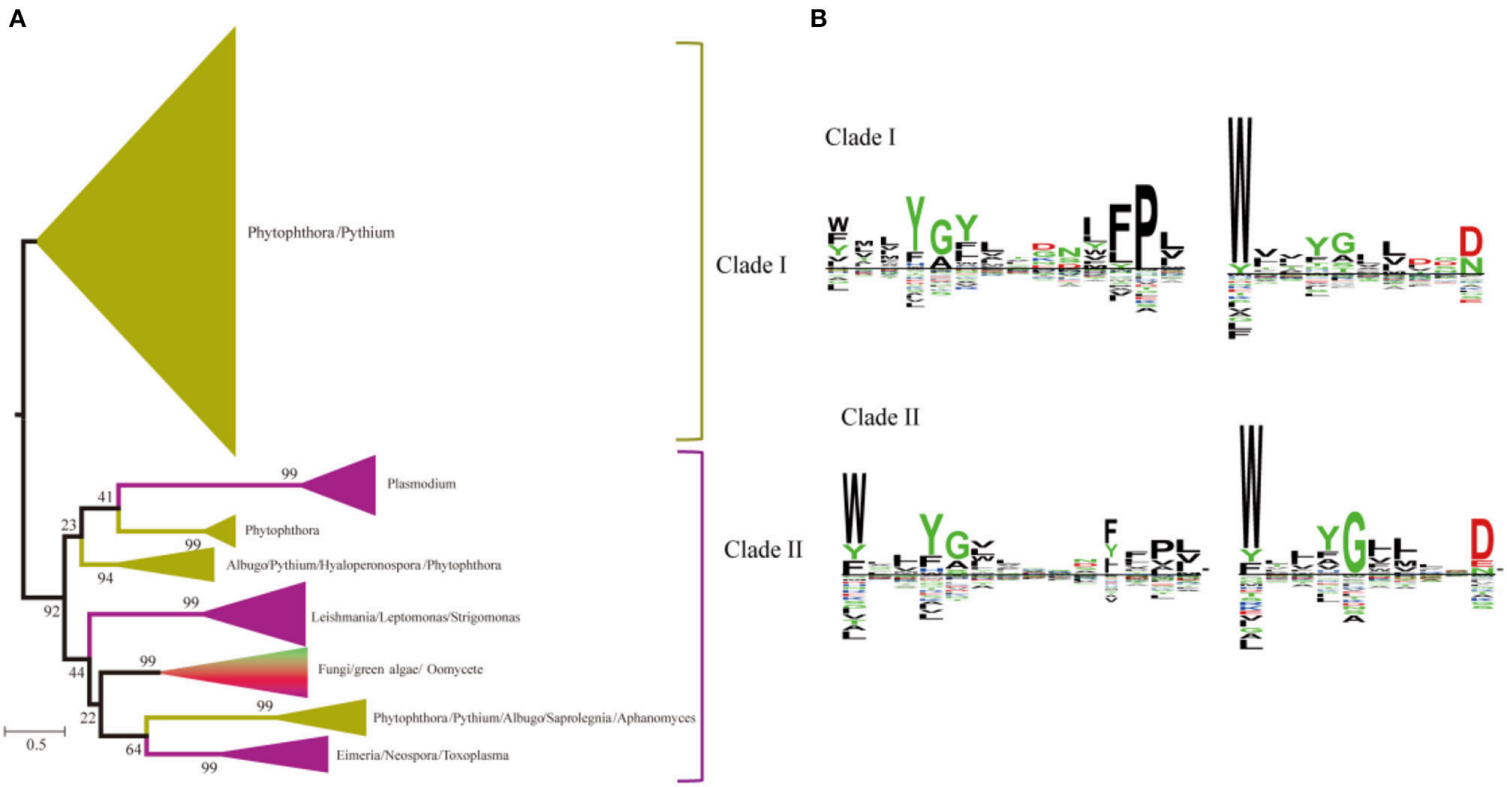
Phylogenetic and conserved motif analyses of SWEETs from unicellular eukaryotes and fungi. **(A)** Molecular phylogenetic analysis by the maximum likelihood method. The tree with the highest log likelihood (−116121.8598) is shown. The tree is drawn to scale, with branch lengths indicating the number of substitutions per site. Evolutionary analyses were conducted in MEGA7. **(B)** Sequence alignment of conserved motifs of SWEETs in different clades corresponding to **(A)**. The sequences were aligned by ClustalW. The conservation level of each residue is indicated by the height of the bar above it. The sequence logo was generated using Seq2Logo.

A heterogeneous distribution of SWEETs is found in the number of copies of each paralog in the different genera of unicellular eukaryotes and fungi (Abascal et al., 2014). Recent studies showed that SWEETs from oomycetes, fungi, and algae clustered as a complex clade separated from the animal and plant clusters, and *Phytophthora* species contain large SWEET clusters (Hu et al., 2016). Two clades of paralogs can be distinguished in unicellular eukaryotes and fungi based on the phylogenetic tree (Figure 6A). Members of the first clade are found only in oomycetes, including *Phytophthora* and *Pythium*. Members of the second clade are found in organisms ranging from oomycetes to green algae. Both clades have unicellular eukaryotes and fungi with C-terminal WX_n_D motifs. In the N-termini of the two clades, clade I has a conserved Pro in the conserved motif, while Trp is much conserved in clade II (Figure 6B). The bulk of SWEET expansion occurs in the oomycetes. The oomycete *Phytophthora parasitica* is a soil-borne pathogen with a wide range of hosts and represents most species in the genus *Phytophthora* (Meng et al., 2014). In *P. parasitica* strain INRA-310, which has an available draft genome sequence, 26 homologs of SWEETs with low sequence identity can be identified by the presence of the PQ loop (Supplemental Figure 2).

### Distribution and phylogenetic analysis of SemiSWEETs in prokaryotes

**Figure 7:**
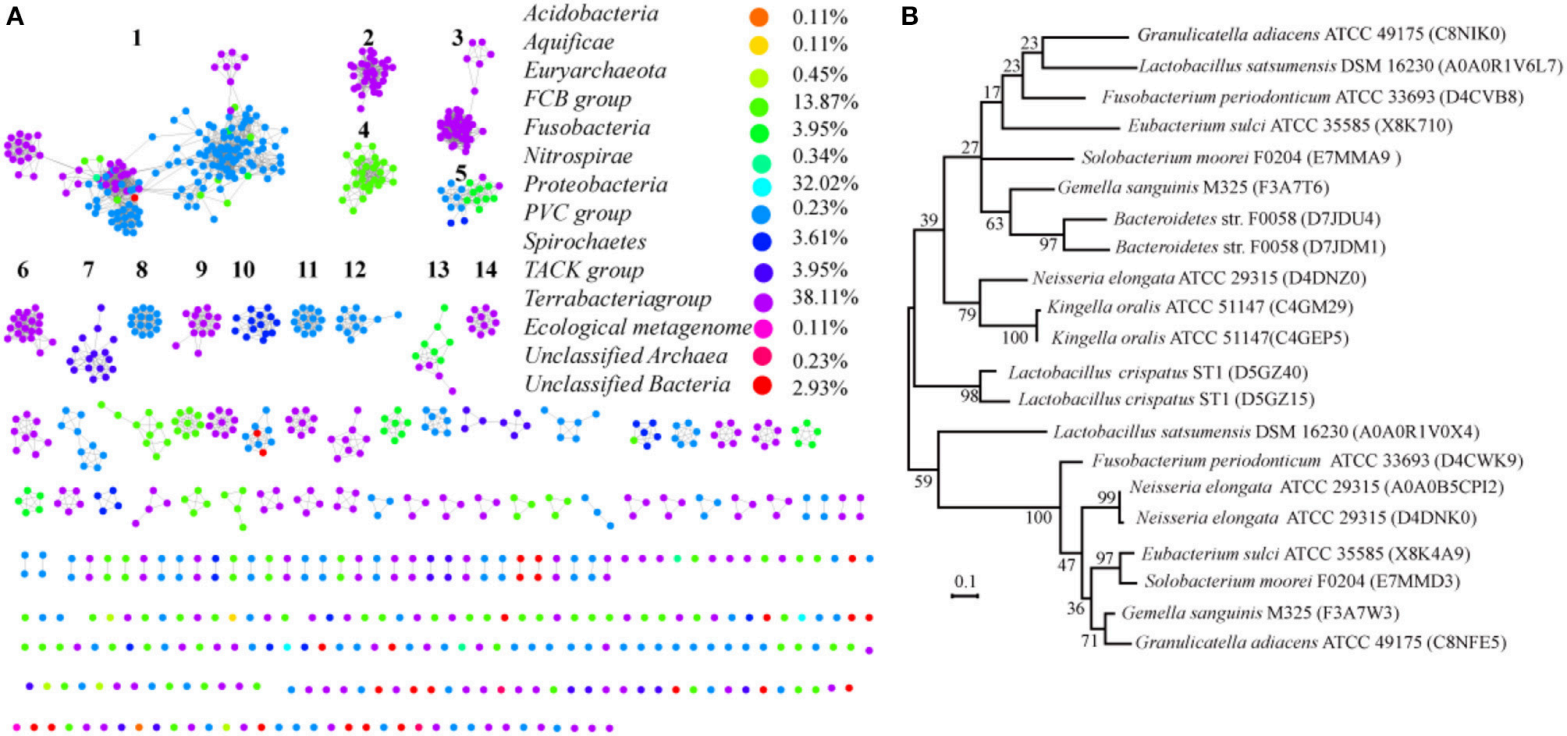
Taxonomic distribution and evolution of SemiSWEETs in prokaryotes. **(A)** Distribution of SemiSWEETs. Each node represents one protein. Edges are shown with BLASTP e-values below the indicated cutoff. Clusters with more than 10 nodes are labeled sequentially. Nodes from the same taxonomic groups in the global network are the same color. The colors corresponding to the different phyla and the percentage of proteins in each phylum are listed on the right. **(B)** Molecular phylogenetic analysis of SemiSWEETs from bacteria with >1 copy of the gene by the maximum likelihood method. The tree with the highest log likelihood (−2328.2531) is shown. The percentages of replicate trees in which the associated taxa clustered together in the bootstrap test (1,000 replicates) are shown next to the branches.

Most of the proteins from prokaryotes can be clustered into 14 groups with at least 10 proteins, and each protein was painted according to its taxonomic classification (Figure 7A). Members of the SemiSWEET family are found in 11 phyla or superphyla in the domains Bacteria and Archaea. The relative abundance of SemiSWEET proteins varies widely among phyla. The prevalence of *SemiSWEET* genes is high in *Terrabacteria*, which accounts for 38.1% of all *SemiSWEET* genes examined. *Proteobacteria* shows the second highest abundance (32.0%), and the presence of SemiSWEETs in other phyla is limited. To provide a more detailed view of the evolutionary relationships across the groups, a phylogenetic analysis was performed using the proteins in the 14 clusters that had been assigned based on sequence comparisons (Supplemental Figure 3). The proteins from clusters 1, 8, and 10 form a clade in the phylogenetic tree. The proteins from archaea (cluster 7) are most similar to the proteins of other bacteria and form another clade. Furthermore, it was previously reported that no prokaryote had been found to have more than 1 SemiSWEET (Hu et al., 2016). Our research showed that there are several strains containing 2 SemiSWEETs, and 3 SemiSWEETs occur in *Neisseria elongata* ATCC 29315 (Figure 7B). In the strains with 2 SemiSWEETs, the sequences of the two proteins are similar and located close together in the phylogenetic tree, such as the proteins from *Kingella oralis* ATCC 51147. On the other hand, the two SemiSWEETs from *Granulicatella adiacens* ATCC 49175 show lower identity with each other and are located in different clusters. In *N. elongata* ATCC 29315, two SemiSWEETs are close to each other while in the third is in another cluster (Figure 7B). These results suggest that both gene fusion and horizontal gene transfer occurred during evolution of SemiSWEETs in bacteria.

### Consensus and coevolution of amino acids in SWEETs

**Figure 8:**
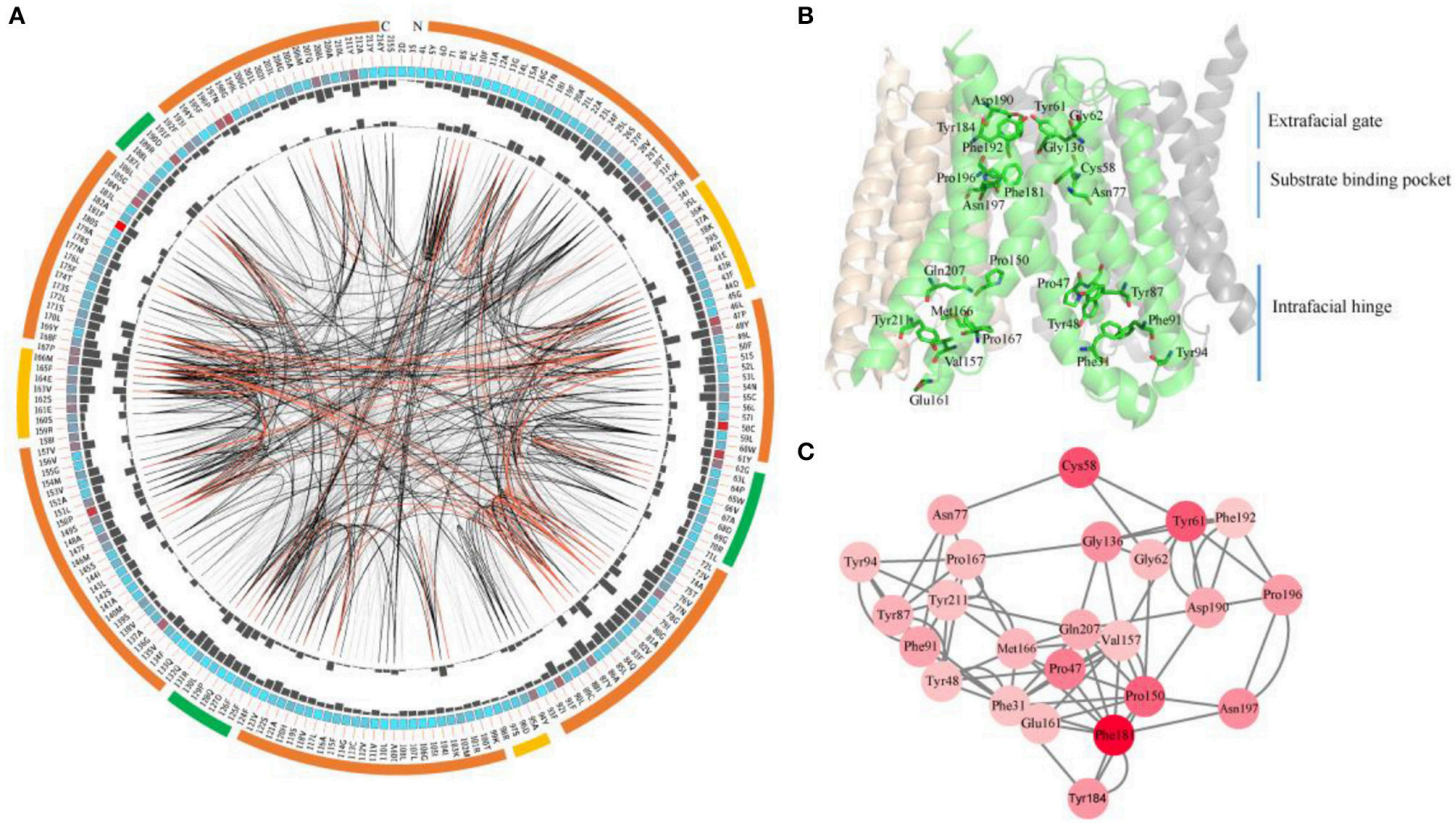
Conserved and coevolved residues in SWEETs represented using OsSWEET2b as a reference sequence. **(A)** Network analysis of conserved and coevolving residues. The circular network shows the connectivity of coevolving residues. The outermost circle represents the TMHs (orange), the intrafacial region (yellow), and the extrafacial region (green). The labels in the second circle indicate the alignment positions and amino acids of OsSWEET. The colored square boxes in the second circle indicate MSA position conservation (highly conserved positions are shown in red and less conserved positions in blue). The third and fourth circles show the proximity mutual information (MI) and cumulative MI (cMI) values as histograms facing inward and outward, respectively. In the center of the circle, the edges that connect pairs of positions represent significant MI values (>6.5), with red lines indicating the highest MI scores (top 5%), black lines indicating midrange scores (between 70 and 95%), and gray lines indicating the lowest scores (the remaining 70%) as defined by MISTIC. **(B)** Ribbon diagram of OsSWEET2b (PDB ID: 5ctg) showing the 24 most coevolved and conserved residues. **(C)** The network cMI with high conservation value. Nodes represent the 24 most conserved residues (labeled with position and code) and nodes are colored to indicate conservation, from red (higher) to pink (lower). The length of each edge is inversely proportional to its MI value (the closest nodes have the highest MI values).

Since most SWEETs contain 6-7 TMHs, the sequences of SWEETs with 6-7 TMHs were used to determine consensus sites, allowing examination of the conservation of the primary sequences of SWEETs. The protein sequence of OsSWEET2b (Q5N8J1) was used as the reference sequence to create and display the MSAs, and the conservation of the residues is shown in Figure 8A and Table 1. The highly conserved residues were mapped onto the structure of OsSWEET2b (Figure 8B) and further analyzed to determine amino acid coevolution (Figure 8C).

**Table 1:** Effects of highly conserved residues on the activity of SWEET.

**Ranking**	**Relative conservation^a^**	**Position in OsSWEET2b**		**Position in AtSWEET1**		**Mutated amino acids**	**Impact on activity**	**Origin**
1	100	181	F	176	W	F	Abolish	Tao et al., 2015
2	77	58	C	54	S	A or C	No effect	Tao et al., 2015
3	74	61	Y	57	Y	A	Abolish	Xuan et al., 2013
4	71	150	P	145	P	A	Abolish	Tao et al., 2015
5	64	47	P	43	P	A	Abolish	Tao et al., 2015
6	59	197	N	192	N	A	Abolish	Tao et al., 2015
7	58	136	G	131	G	D	Abolish	This study
8	57	184	Y	179	Y	A	Reduce	Xuan et al., 2013
9	55	196	P	191	P	T	Abolish	This study
10	52	91	F	87	F	A	Abolish	This study
11	51	190	D	185	D	A	No effect	Tao et al., 2015
12	50	77	N	73	N	A	Abolish	Tao et al., 2015
13	50	207	Q	202	Q	D	Abolish	This study
14	49	87	Y	83	Y	A	Reduce	This study
15	48	166	M	161	M	A	Abolish	This study
16	46	62	G	58	G	D	Abolish	Xuan et al., 2013
17	45	167	P	162	P	A	Abolish	Tao et al., 2015
18	45	161	E	156	K	R	No effect	This study
19	45	94	Y	90	Y	A	No effect	This study

a *The conservation value is calculated based on ClustalW. The highest conserved amino acid is set to 100*.

OsSWEET2b forms a homotrimer, and each protomer consists of 7 TMHs with an N-terminal domain (the first 4 TMHs) and a C-terminal domain (the last 3 TMHs) (Tao et al., 2015). In TMH1, one proline residue at the position equivalent to the PQ motif in SemiSWEETs is conserved (Figure 8A). The proline serving as a flexible hinge is conserved in both SWEETs and SemiSWEETs, but glutamine is conserved only in the SemiSWEETs. Furthermore, substitution of glutamine with alanine slightly affects the activity of SemiSWEETs (Lee et al., 2015). In TMH2, a cysteine residue is highly conserved. This cysteine, together with the highly conserved Asn77 in TMH3, Phe181 in TMH6, and Pro196 and Asn197 in TMH7, forms a key cluster for the substrate binding pocket (Figures 8A,B). The second conserved residue in TMH2 is Tyr61. It has been reported that Tyr61-Asp190 are the major constituents of the extrafacial gate (facing the vacuolar lumen) of the protein (Tao et al., 2015). Our results indicate that Gly62, Gly136, Tyr184, and Phe192, which surround the extrafacial gate, are highly conserved (Figure 8B; Table 1). The second conserved residue in TMH2 is Pro47, which is in an intrafacial hinge point. Other proline residues that may function as hinge points, including Pro150 and Pro167, are also conserved. The intrafacial (cytosolic) gate is required for sugar transport, and mutations in the conserved prolines that are important for the intrafacial gate of AtSWEET1 (SWEET1 from *Arabidopsis thaliana*) abolish its glucose transport activity (Tao et al., 2015). Near the intrafacial side, TMH1, TMH2, TMH5, and TMH6 are the main constituents of the transport route. MSA results show that Phe31, Tyr48, Tyr87, Phe91, Tyr94, Val157, Glu161 Met166, Gln207, and Tyr211 in the TMHs are highly conserved and located near the putative intrafacial gate (Figure 8A). Further mapping of the most highly conserved amino acids in the structure of OsSWEET2b revealed that these amino acids form three conserved clusters, which may function as the extrafacial gate, substrate binding site, and intrafacial gate. Each conserved residue belongs to a cluster: 6 to the extrafacial gate, 6 to the substrate binding pocket, and 13 to the intrafacial gate (Figure 8B).

Because a combination of changes in SWEET sequences might have directed novelties in evolution, the coevolution of SWEET amino acids was investigated using MI (Figures 8A,C). If two residues share a high MI value, they are probably coevolving and to maintain a given function, a mutation of one residue is linked to a specific compensatory mutation of the other residue (Petit et al., 2014). The MI network for SWEET family members revealed that the highest 10% of MI values belong to amino acids in TMH1, TMH2, TMH3, TMH5, and TMH6 (Figure 8B). The most highly conserved residues in SWEETs were further considered for coevolution analysis as shown in Figure 8C. The 24 highest-scoring residues form a connected distance network, indicating that these residues also share a high MI score (Figure 8C). A strong correspondence between coevolving residue positions and conserved residues is consistent with previous studies (Tse and Verkhivker, 2015; Jia et al., 2017b).

### MD simulation

**Figure 9:**
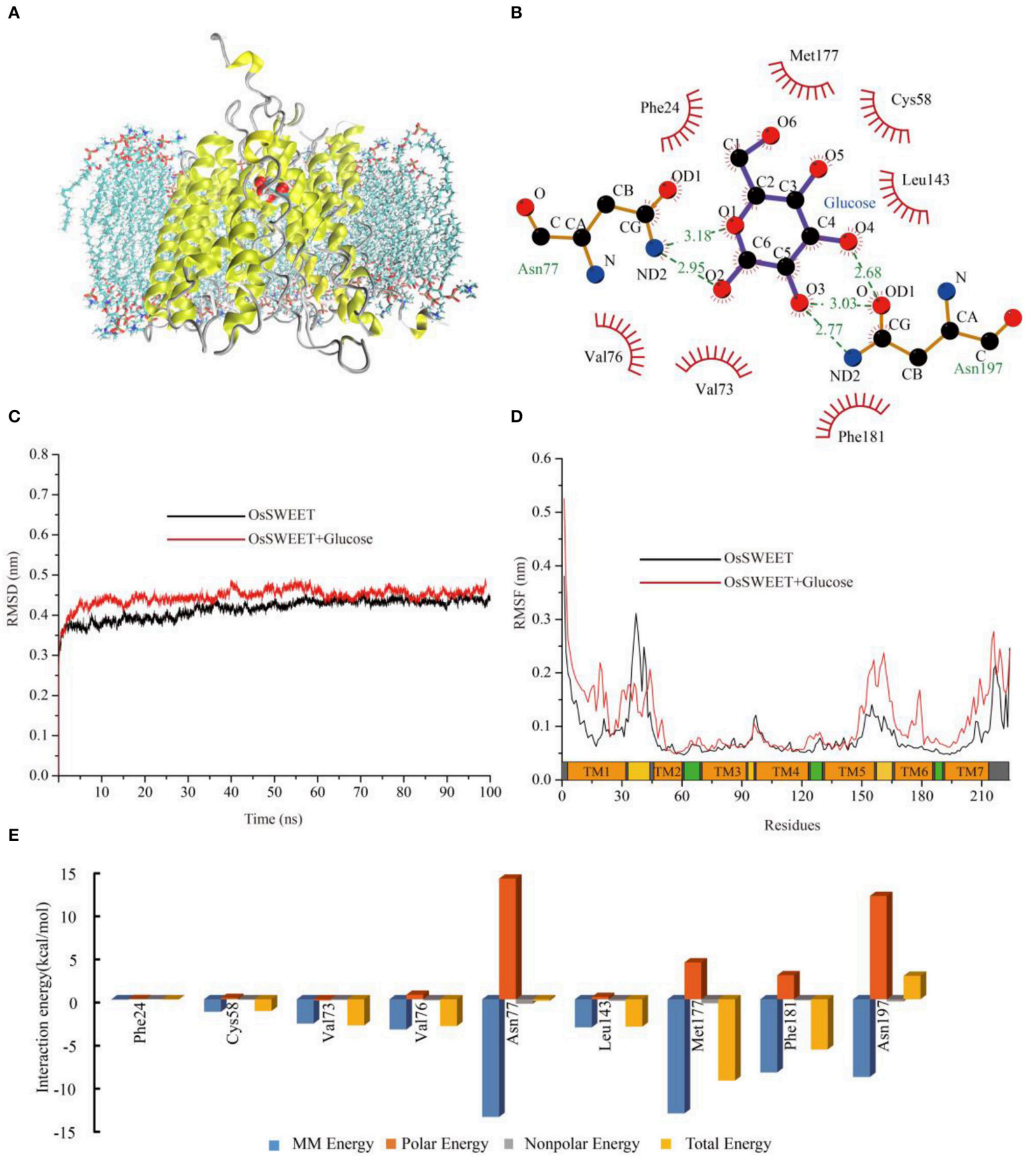
Molecular dynamic analysis of OsSWEET2b. **(A)** Snapshot of a representative simulation system. The protein is shown in ribbons and glucose is shown in ball-and-stick form. **(B)** The glucose binding sites. A two-dimensional representation of ligand-protein interactions for the OsSWEET2b-glucose complex. **(C)** Backbone RMSDs are shown for OsSWEET2b and OsSWEET2b-glucose complexes at 300 K. The black line indicates apo-OsSWEET2b; OsSWEET2b-glucose is shown in red. **(D)** RMSFs of the residue positions of apo-OsSWEET2b and OsSWEET2b-glucose at 300 K. apo-OsSWEET2b and OsSWEET2b-glucose are shown in black and red, respectively. **(E)** The decomposition of the binding energy on a per-residue basis at the binding sites of the OsSWEET2b-glucose complex. (Blue bars: molecular mechanics energy; orange bars: polar energy; gray bars: non-polar energy; yellow bars: total energy).

To explore the potential functions of the conserved amino acids in OsSWEET2b, we used OsSWEET2b and glucose as examples to carry out molecular docking and MD simulations to gain insight into the ligand binding mechanism. The analyses revealed that OsSWEET2b binds glucose using the side chains of Phe24, Cys58, Val73, Val76, Leu143, Met177, and Phe181, which form a hydrophobic pocket around glucose. Glucose also forms hydrogen bonds with Asn77 and Asn197, which are highly conserved within SWEETs (Figure 9B). The two hydrogen bonds between glucose and Asn77 are generated mainly by the amide group of Asn linking with O1 and O2 of glucose. Asn197 can form three hydrogen bonds with glucose: one links the amide group and O3 of glucose, and two others form between the carbonyl group of Asn and O3 and O4 of glucose (Figure 9B).

To gain more information about the binding of glucose by OsSWEET2b, the transporter-ligand structure embedded in a POPC lipid bilayer environment (Figure 9A) was used for MD simulations to investigate the conformational changes and protein internal motions using GROMACS software (Abraham et al., 2015). In the simulation, the root-mean-square deviation (RMSD) of Cα atoms is a crucial parameter of convergence in protein structural changes over the course of a simulation (Figure 9C). The backbone RMSD of apo-OsSWEET2b equilibrates approximately 0.42 nm after 5 ns of simulation. The backbone RMSDs of OsSWEET2b-glucose equilibrates approximately 0.45 nm over the same time frame, as shown in Figure 9C. OsSWEET2b in complex with glucose shows a higher RMSD value than does apo-OsSWEET2b. This suggests that glucose binding causes a conformational change in OsSWEET that increases its flexibility.

In contrast to RMSD, RMSF of Cα atoms was calculated to identify the flexible regions of the protein (Figure 9D). The results of RMSF calculations for apo-OsSWEET2b show that the highest RMSF values belong to the amino acids at the N- and C-termini, which suggests that the two termini are the most flexible regions of OsSWEET2b. Higher flexibility is observed for the internal regions between TMH1 and TMH2 and for the residues between TMH5 and TMH6. Lower flexibility is observed for the TMH segments; this finding is in accordance with the glucose transporter 4 (GLUT4) from human, an insulin-facilitated glucose transporter (Mohan et al., 2010). Binding of glucose causes a difference in the fluctuation patterns of the protein. In the transporter-ligand complex, the TMH1 region, the internal region between TMH5 and TMH6, and the TMH6 and TMH7 regions becomes more flexible after binding to glucose, which is seen in increased RMSF values. In contrast, the internal region between TMH1 and TMH2 becomes more rigid. This result indicates a glucose-mediated conformational rearrangement in these regions.

As discussed above, differences in the conformations and dynamic behaviors were observed between apo-OsSWEET2b and the complex. Quantification of the contributions of the residues to glucose binding could provide further insight into the precise function of each amino acid. Therefore, glucose-residue interaction decomposition was performed by the MM/PBSA method using the g mmpbsa package (Kumari et al., 2014; Genheden and Ryde, 2015). The summations of the total interaction free energies (Δ*G*_*total*_) were separated into molecular mechanics energy (Δ*E*_*MM*_), polar binding energy (Δ*G*_*polar*_), and non-polar solvation free energy (Δ*G*_*np*_). The energy contributions from these residues are summarized in Figure 9E and most of the residues showed the favorable Δ*G*_*total*_ values except Asn197. The energy decomposition results showed that Phe24, Cys58, Val73, Val76, Leu143, Met177, and Phe181 have weak hydrophobic interactions with glucose. Among them, Met177 and Phe181 also have significant Δ*E*_*MM*_ values. The two Asn residues (77 and 197) have significant Δ*E*_*MM*_ values of −13.7 and −9.0 kcal/mol, respectively, but the Δ*G*_*polar*_ values of the two residues are 14.0 and 12.0 kcal/mol, respectively. As a result, the calculated Δ*G*_*total*_ values for the ligands are −0.2 and 2.7 kcal/mol. The unfavorable Δ*G* value of Asn197 binding to glucose suggests that Asn197 interacts with the substrate in an antagonistic fashion.

### Site-directed mutagenesis of AtSWEET1

**Figure 10:**
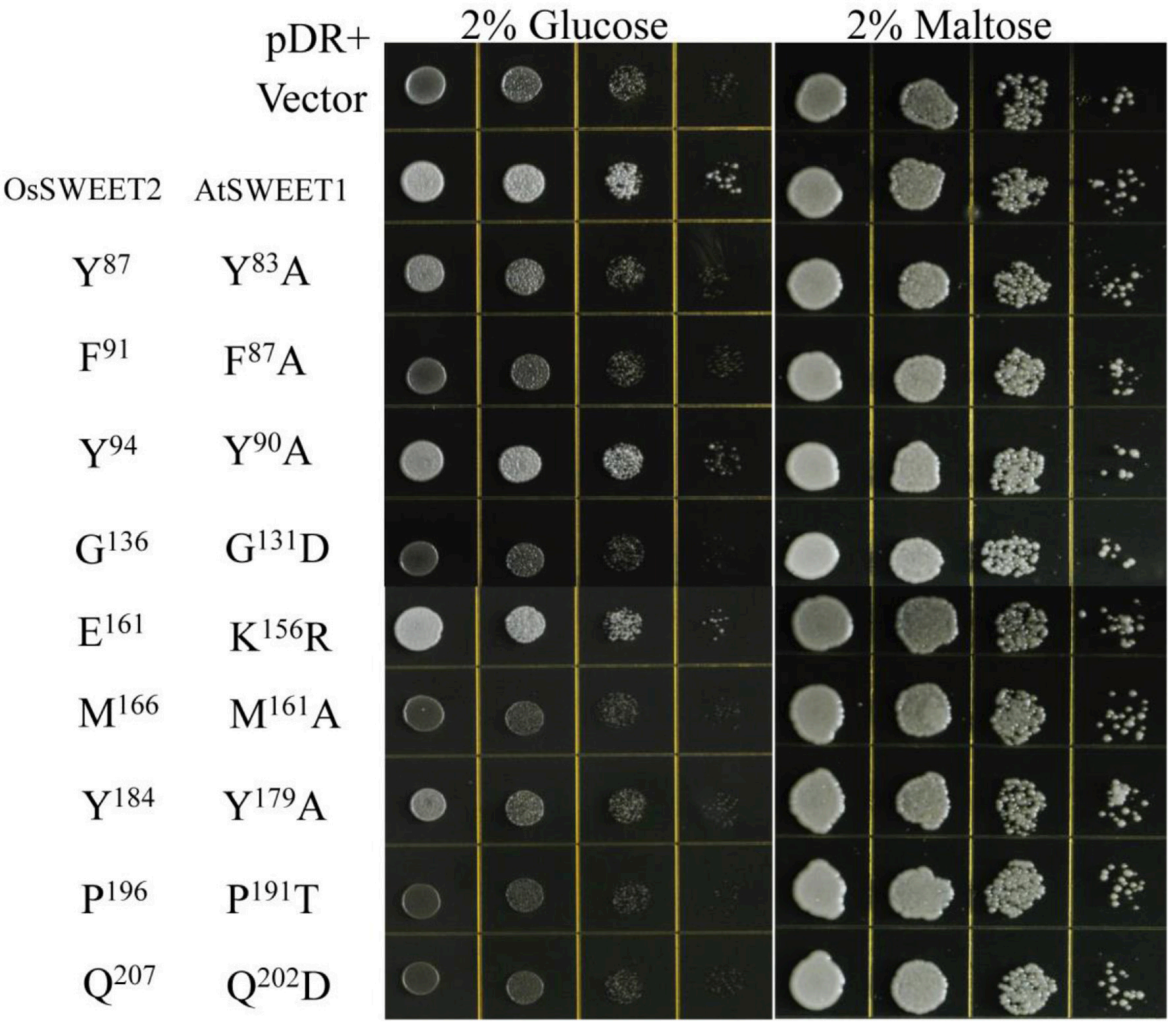
Growth assays of AtSWEET1 mutants expressed in yeast strain EBY4000 were performed on YNB medium containing 2% glucose or maltose. Except for AtSWEET1 mutants Y^83^A, Y^90^A, Y^179^A, and K^156^R, other mutations (F^87^A, G^131^D, M^161^A, P^191^T, and Q^202^D) in AtSWEET1 led to loss of glucose transport activity. Empty vector (pDRf1) and AtSWEET1 were used as the negative and positive controls, respectively. 10^6^ yeast cells were spotted in the starting points and further grown at 28°C for 3 days. The corresponding conserved residues in OsSWEET2 are shown next to the residues from AtSWEET1.

The amino acid conservation and MD simulation analyses reveal that the highly conserved residues are involved in sugar transport. The functions of some conserved amino acids have been studied previously in *A. thaliana* SWEET1 (AtSWEET1) (Table 1; Xuan et al., 2013; Tao et al., 2015). These studies showed that replacing the amino acids located in the extrafacial gate (Y57A, G58D, and V188A), the substrate binding pocket (N73A, N192A, and W176A), and the intrafacial gate (P23A or P23T, P43A, P145A, and P162A) abolished activity. However, substitution of Ser54 in the substrate binding pocket with alanine or cysteine and replacement of Tyr179 or Asp185 at the extrafacial gate with alanine reduced transport activity of AtSWEET1 (Table 1). To further confirm the necessity of the conserved residues for protein function, the other eight highly conserved residues were mutated in AtSWEET1, and glucose transport activity was examined in hexose-transport-defective yeast strain EBY4000 (Table 1; Figure 10). Among these residues, Gly131 and Pro191 are located in the extrafacial gate and the substrate binding site, respectively, whereas 6 other residues are in the intrafacial gate. Replacing the residues located in the extrafacial gate and the substrate binding site abolishes AtSWEET1 glucose transport activity. Mutation of F87A, Q202D, and M161A, in the intrafacial gate, also fully abolishes the activity. The Y83A mutant exhibits partially reduced activity, and the K156R and Y90A mutants, which have mutations in the less conserved residues, show no change in AtSWEET1 activity. The MD and mutagenesis results confirm that the conserved amino acids occupying significant positions in the structure play crucial roles in binding and transporting sugars.

## Discussion

In this study, we first performed a large-scale *in silico* analysis of SWEETs and SemiSWEETs, which revealed that these transporters are widely distributed in archaea, bacteria, and eukaryotes. The evolutionary relationships of these proteins were assessed by SSN and phylogenetic tree analyses, showing that gene fusion, duplication and horizontal gene transfer have been the critical forces driving evolution. Sequence analyses and point mutation-based activity tests indicate that the residues that are directly involved in sugar binding and transport are highly conserved and have coevolved. These residues bind sugars by hydrophobic interactions and hydrogen bonds.

SWEETs and SemiSWEETs are particularly abundant in plants, followed by bacteria, with the explosive growth of bacterial genome sequences (Land et al., 2015). This result is consistent with a previous study, which showed that SemiSWEETs occur in only a limited number of prokaryotes (Xuan et al., 2013). The uneven distribution of SWEETs in the biosphere suggests that the functions of these proteins differ among organisms. SWEETs are mainly responsible for the efflux of sugars. Sugar efflux is important for plant nectar production, grain filling and pollen development. Therefore, it is expected that the majority of SWEET or SemiSWEETs (44.4%) be observed in green plants, the unique multicellular organisms that possess photosynthetic activity and complicated sugar translocation processes. Sugar efflux transporters are also essential for the maintenance of animal blood glucose levels, and HsSWEET1 in human may be involved in this process (Chen et al., 2010). SemiSWEET from *Streptococcus* was suggested to transport a product of glucosidase because it is located in the same operon as a putative 6-phospho-β-glucosidase (Xuan et al., 2013), while SemiSWEET (F0T9U2) from *Methanobacterium lacus* (strain AL-21) is associated with a trehalose-phosphatase, an enzyme involved in trehalose synthesis. This SemiSWEET may mediate the efflux of compatible solutes. It seems that SemiSWEETs may have a broad range of substrates. An abundance of SWEETs is also found in oomycetes. More than 60% of the known oomycetes are plant parasites, able to absorb nutrients directly from plants (Thines and Kamoun, 2010). SWEETs in oomycetes may uptake sugar from the interface between the oomycetes and hosts. We also speculate that SWEETs in oomycetes may have roles in efflux of molecules such as phytoalexin, enlightened by an observation that an ABC transporter from *Botrytis cinerea* exports phytoalexin, a virulence factor in *A. thaliana* (Stefanato et al., 2009). Taken together, we propose that SWEETs may be able to transport a wide range of substrates.

Yee et al. showed that SWEETs with other transporter, opsin, and G protein-coupled receptor may evolve via duplication of a 4 TMH basic unit followed by loss of an N-terminal (more frequent) or C-terminal TMS (less frequent) (Yee et al., 2013). Currently, there are two mechanisms that may explain the evolution of SWEETs: (1) SWEET was generated by the duplication and fusion of a SemiSWEET (Xuan et al., 2013); and (2) SWEET was generated by the fusion of an archaeal and a bacterial SemiSWEET (Hu et al., 2016). Both mechanisms suggest that gene fusion is a necessary step for SWEET formation. Previously, we found that AtSWEET11 exhibited much stronger activity than BjSemiSWEET in uptake and efflux of sucrose (Xuan et al., 2013), suggesting that the number of TMHs may be linked to the transport activity. To form transport pore, the dimerization of SemiSWEETs is required in bacteria (Xu et al., 2014), but SWEETs with 7 TMHs are still not able to make a big enough pore and they require the trimeric structure (Tao et al., 2015). Therefore, duplication and fusion of SemiSWEET and SWEET could be an efficient way to create a functional translocation pore evolutionally, which may lead to a higher transport efficiency with a significant impact on evolution. Our analyses show that SWEETs with 6 or 7 TMHs are also found in bacteria, such as the SWEET from *Alteromonadales* TW-7 (Figure 3). The first 3-TMH unit and the second unit of the SWEET show high similarity to the two semiSWEETs from *Legionella brunensis*, respectively (Supplemental Figure 4). The results suggest that duplication and fusion of SemiSWEETs can occur in bacteria itself. Interestingly, more than 100 SemiSWEETs with 3 or 4 TMHs were identified in plant genomes, also suggesting that SWEETs may be fused in plants from SemiSWEETs with 3 or 4 TMHs directly.

The one extraSWEET protein from *V. vinifera* that possesses 14 TMHs might have arisen from an internal duplication of a 7-TMH SWEET (Patil et al., 2015). Our research showed one 15-TMH SWEET (OpSWEET) occurs in a wild rice (*O. punctata*) genome. Amino acid sequence alignments showed that the 15-TMH SWEET shared high sequence identity with the fusion forms of OsSWEET6a and OsSWEET6b (Supplemental Figure 5). Whether and how OpSWEET is formed from the fusion of OsSWEET6a and OsSWEET6b remain to be further explored. Furthermore, superSWEETs with >18 TMHs are found in oomycetes, among which, a SWEET from *Phytophthora ramorum* (H3GD93) has 25 TMHs, indicating that the protein formed from the fusion of 4 SWEETs (Figure 3). Each of these 4 units shows >70% identity to a 7-TMH SWEET from *Phytophthora* species (Supplemental Figure 6). The increased number of SWEET units and the sequence similarity indicate that gene duplication and fusion is a common mechanism during the evolution of SWEETs, which may lead to sub-functionalization (i.e., functional split) and neo-functionalization (i.e., new function) (Andersson et al., 2015).

Recently, research showed that whole-genome duplication, or segmental duplication and dispersed duplication, played important roles in SWEET family evolution in six species of pear (Li et al., 2017). Plant genomes generally contain more than 10 copies of SWEETs (Yuan and Wang, 2013), and a copy number >1 is also observed in Nematoda and Insecta but not in Teleostomi. Our recent study showed that the wheat harbors 59 SWEET genes (Gao et al., 2018). Patil et al. identified 52 SWEET genes in the genome of the soybean (Patil et al., 2015). Currently, the two crops represent the top two highest copy numbers of SWEET genes across the biosphere. The large copy numbers might be due to the whole genome duplication during evolution/domestication process. Interestingly, multiple copies of SWEETs also occur in oomycetes (Supplemental Figure 2). Considering the copy numbers and numbers of TMHs in oomycetes, both gene fusion and gene duplication appear to contribute to the evolution of SWEETs in oomycetes. Finally, it has been reported that bacteria have only one SWEET gene (Yuan and Wang, 2013; Hu et al., 2016). Mining of the SWEET database reveals the existence of bacteria, but not archaea, with >1 SemiSWEET. The SemiSWEETs in these strains can have very similar sequences or show low identities. The functions of these SemiSWEETs need to be tested, but their copy numbers and sequence characteristics suggest that horizontal transfer of SemiSWEET genes occurred between bacteria.

According to the analysis of conserved residues in SWEETs, several residues located in the three important areas of the structure are highly conserved. Of these 19 residues, mutations at 4 (S54A, Y90A, K156R, and D185A) do not affect AtSWEET1 activity. However, mutations of Y83A and Y179A partially abolish, while mutations of the other 13 residues fully abolish, AtSWEET1 glucose transport activity (Table 1; Figure 10). Cys58 in the putative binding pocket is highly conserved in OsSWEET2b, but replacing its equivalent residue, S54, in AtSWEET1 has little effect on transport activity. This may be explained if the substitution of Ser with Cys or Ala does not affect the contribution of the amino acid to the binding of glucose, as the MD analysis indicates that the residue binds the substrate by hydrophobic interaction (Figure 9). The three other mutations (Y90A, K156R, and D185A) in AtSWEET1 do not affect the activity. Y90 and K156 show relatively low conservation values, suggesting that they are not as important as other residues. Mutation of Asp185, located in the extrafacial gate, to Ala does not lead to loss of activity. The role of this residue requires further analysis. Further MD simulation indicates that the conserved residues are always located in less flexible regions of OsSWEET2b irrespective of the presence or absence of bound glucose (Figure 9D). The non-flexibility suggests that these residues play important roles in maintaining protein structure. On the other hand, the presence of glucose in the binding pocket increases the flexibility of the entire OsSWEET2b molecule, mainly due to changes in the TMH1 region and in the region from TMH5 to TMH6. Unlike soluble enzymes that have reduced flexibility after binding substrates (Jia et al., 2017a), the binding of substrates to transporters always increases the flexibility of the proteins. This is seen in the biotin transporter of *Rhodobacter capsulatus* (Finkenwirth et al., 2015), an amino acid antiporter of *Bacillus subtilis* (Bippes et al., 2009) and lactose permease of *E. coli* (Serdiuk et al., 2014). The enhanced conformational flexibility may be required for transporters to allow substrate binding on one side of membrane and facilitate substrate translocation and release on the other side. Though the flexibility of OsSWEET2b increases after binding glucose, the amino acids in the binding pocket still form hydrophobic interactions in a non-polar pocket, and Asn77 and Asn197 interact with the substrate via hydrogen bonds. This is similar to SemiSWEET from *Leptospira biflexa* forming hydrogen bonds with glucose via Asn64 for substrate translocation (Latorraca et al., 2017). Among the residues interacting with glucose in OsSWEET, only Asn197 has a glucose interaction with a positive binding free energy (Δ*G*), indicating that the binding of Asn197 to glucose is not stable. Together with the flexible mechanism of OsSWEET2b, this suggests that Asn197 plays a crucial role in the translocation of glucose.

In summary, our results indicate that SWEETs, SemiSWEETs, and their homologous proteins exist in both prokaryotes and eukaryotes; in particular, many SWEETs are found in oomycetes. During the evolution and distribution of SWEETs, gene fusion and gene duplication have been the main driving forces, and horizontal transfer of these genes also occurred in bacteria. The key residues involved in sugar translocation by SWEETs were conserved during evolution. The conformational changes of different regions during sugar binding are also illustrated.

## Author contributions

YX, BJ, and CJ conceived and designed the sequence data analysis, and wrote the manuscript. XZ performed the mutation analyses. ZP performed the MD analyses. YD, LH, and JZ contributed to data analysis. L-QC reviewed and edited the manuscript. All authors read and approved the final manuscript.

### Conflict of interest statement

The authors declare that the research was conducted in the absence of any commercial or financial relationships that could be construed as a potential conflict of interest.
