# Supplementary material for: Integrative View of the Diversity and Evolution of SWEET and SemiSWEET Sugar Transporters
Source: Front Plant Sci. 2017 Dec 20;8:2178. doi: 10.3389/fpls.2017.02178 (PMC5742349; doi:10.3389/fpls.2017.02178)
Supplement: Supplementary file 1 [file Presentation1.PDF]

Supplemental Table 1. Primer sequences used in this study.

Primer	Sequence
AtSWEET1 GW-F	GGGGACAAGTTTGTACAAAAAAGCAGGCTTCGGTACC ATGAACATCGCTCACACTATC
AtSWEET1 GW-R	GGGGACCACTTTGTACAAGAAAGCTGGGTGACTAGTA ACTTGAAGGTCTTGCTTTCC
SWEET1 Y83-F	GTGATCGAAACAGTCGCCGTG
SWEET1 Y83-R	CACGGCGACTGTTTCGATCAC
SWEET1 F87-F	TGTTAATCGCTCTTTTCTACG
SWEET1 F87-R	CGTAGAAAAGAGCGATTAACA
SWEET1 Y90-F	CTTTTCGCCGCACCAAAGAAG
SWEET1 Y90-R	CTTCTTTGGTGCGGCGAAAAG
SWEET1 G131-F	TTTTGTGATCTCGCAGCTACT
SWEET1 G131-R	AGTAGCTGCGAGATCACAAAA
SWEET1 K156-F	GTAAAGACGCGGAGTGTAGAG
SWEET1 K156-R	CTCTACACTCCGCGTCTTTAC
SWEET1 M161-F	AGTGTAGAGTTTGCGCCATTC
SWEET1 M161-R	GAATGGCGCAAACTCTACACT
SWEET1 Y179-F	TGGTTCGTCGCTGGTCTAATC
SWEET1 Y179-R	GATTAGACCAGCGACGAACCA
SWEET1 P191-F	GTTGCAATCACAAATGGGTTT
SWEET1 P191-R	AAACCCATTTGTGATTGCAAC
SWEET1 Q202-F	GGGACACTGGACTTGATACTA
SWEET1 Q202-R	TAGTATCAAGTCCAGTGTCCC

Supplemental Table 2. Numbers of SWEETs and SemiSWEETs containing different transmembrane helixes.

TMHs	3	4	5	6	7	8	9	10	12	13	14	15	18	23	25
Archaea	40														
Bacteria	766	14	2	4	15										
Fungi		1			9										
Plantae	47	63	114	249	945	21	3		1	1		2			
Metazoa	19	28	48	118	195	3	1								
Oomycota	19	16	67	145	162	1	1	2	3	1			1	1	1
Algae		2	4	12	23										
Protista	3	5	8	12	40	6	5								

Supplemental Fig. 1

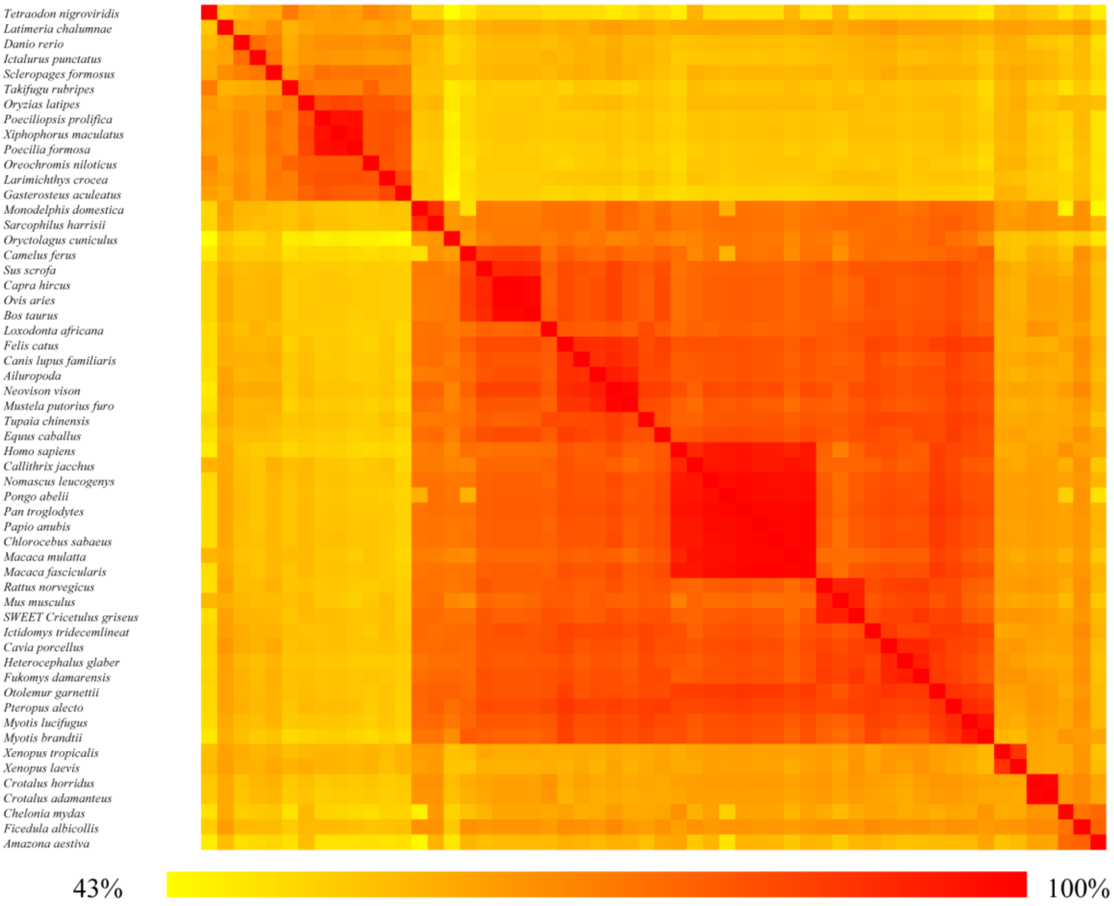

Figure S1. Heat map representation of protein sequence identity of the SWEETs from *Teleostomi* analyzed by ClustalW. The color bar indicates a range of protein sequence identity (43–100%).

Supplemental Fig. 2

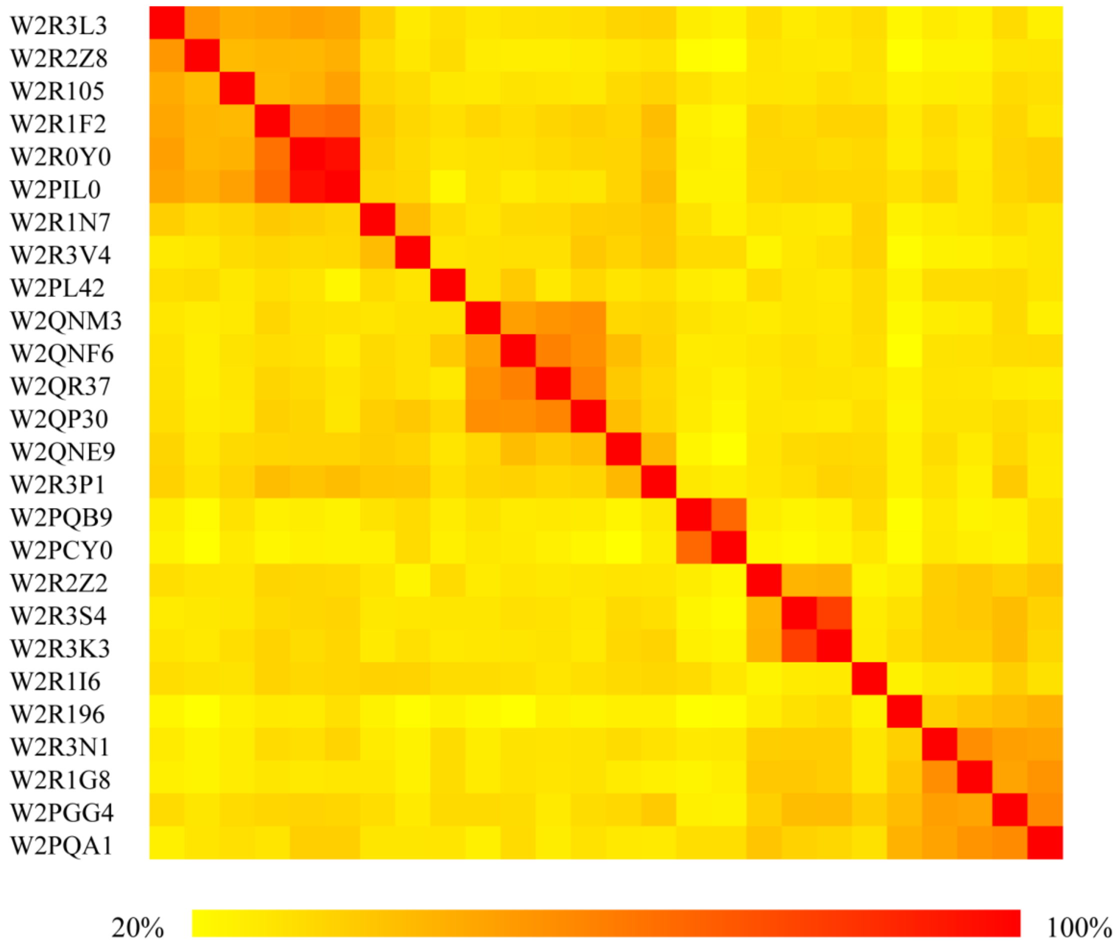

Figure S2. Heat map representation of protein sequence identity of the SWEETs from *Phytophthora parasitica* strain INRA-310 analyzed by ClustalW. The color bar indicates a range of protein sequence identity (20–100%).

Supplemental Fig. S3

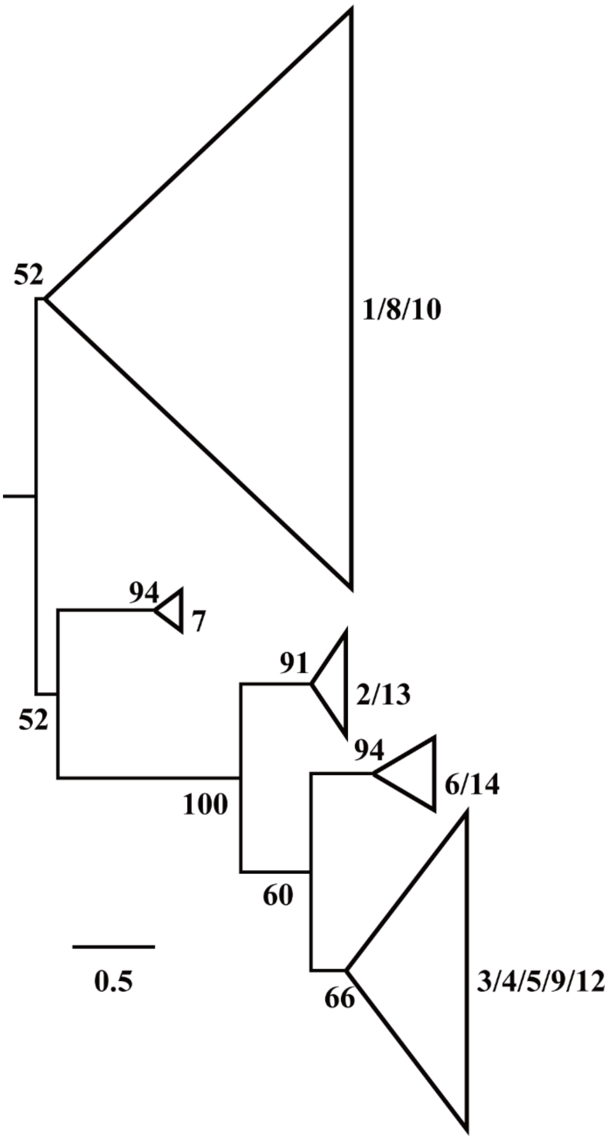

Figure S3. Maximum likelihood phylogenetic tree for SemiSWEETs from bacteria and archaea generated using MEGA7. The tree with the highest log likelihood (-31889.3999) is shown. The percentages of replicate trees in which the associated taxa clustered together in the bootstrap test (1000 replicates) are shown next to the branches.

Supplemental Fig. 4

```
A0Y0J1     : -MAHYLPILSSFILLFSFLPLLNKIRKNRSVSGLSLLMMTFGVAQSLYFITYNIYFERYFIALPFFVTGVLS :  71
A0A0W0S4C9 : MIANLMLIIATILLIISFIPFIKKILNTKSSKGISLTMMMLGILQSISFILHDLYFARYMMVIPFAVITIFF :  72
A0A0W0S4T4 : ------------------------------------------------------------------------ :   -

A0Y0J1     : GLILYFFARYNAAKQERLQLVLMLGFSLMPFSLLLSPNFDTAWVLNALTYVGLVMSSVRVMPQTYKTLRTGD : 143
A0A0W0S4C9 : ITLSILTIKYR------------------------------------------------------------- :  83
A0A0W0S4T4 : ----------------------------------------MDFIFNSICYFGLICGALRVLPQTYKTLRTKE :  32

A0Y0J1     : VSNLSARYFSLQFIAGICGLVVELTNTTPSTSHLLMFIMLLLTNAVQFGCMQYYKMRPIMA----- : 204
A0A0W0S4C9 : ------------------------------------------------------------------ :   -
A0A0W0S4T4 : IDNISLSFFTCHFFAGICGLIYEFTAIKPSIPHFLFFCMVILTNFMQTAYTLYLKKNKLVKECSEL :  98
```

Figure S4. Sequence alignment of SWEET (A0Y0J1) from *Alteromonadales* TW-7 and two SemiSWEETs (A0A0W0S4C9 and A0A0W0S4T4) from *Legionella brunensis*.

Supplemental Fig. 5

```
OpSWEET    : MISPDAARNVVGIIGNVISFGLFLSPVPTFWRICKKKDVEEFKADPYLATLLNCMLWVFYGIPVVHPNSI :  70
OsSWEET6a : MISPDAARNVVGIIGNVISFGLFLAPVPTFWRICKRKDVEEFKADPYLATLLNCMLWVFYGIPVVHPNSI :  70
OsSWEET6b : ---------------------------------------------------------------------- :   -

OpSWEET    : LVVTINGIGLVVEGAYLIIFFLYSPNKKRLRMMAVLGVELVFMVAVILGVLLGAHTHEKRSMIVGILCVF : 140
OsSWEET6a : LVVTINGIGLLVEGTYLLIFFLYSPNKKRLRMCAVLGVELVFMLAVILGVLLGAHTHEKRSMIVGILCVF : 140
OsSWEET6b : ---------------------------------------------------------------------- :   -

OpSWEET    : FGSIMYFSPLTIMGKVIKTKSVEYMPFFLSLVCFLNGVCWTAYALIRFDIYVTIPNGLGAIFGAFQLILY : 210
OsSWEET6a : FGSIMYFSPLTIMGKVIKTKSVEYMPFFLSLVCFLNGVCWTAYALIRFDIYVTIPNGLGALFGAIQLILY : 210
OsSWEET6b : ---------------------------------------------------------------------- :   -

OpSWEET    : ACYYRTTPKKTKSAADKDNVELPSVV-SGPGAANTGSVVSV-TPSISPFLSISLSFIFVRHRREIKMISP : 278
OsSWEET6a : ACYYRTTPKKTKAAKDV---EMPSVVVSGTGAAAAAGGNTGGGSV--------------SVTVER---- : 259
OsSWEET6b : ------------------------------------------------------------------MISP :   4

OpSWEET    : DAARNVVGIIGNVISFGLFLSPVPTFWRICKRKDVEEFKADPYLATLLNCMLWVFYGIPVVHPNSILVVT : 348
OsSWEET6a : ---------------------------------------------------------------------- :   -
OsSWEET6b : DAARNVVGIIGNVISFGLFLSPVPTFWRICKRKDVEQFKADPYLATLLNCMLWVFYGIPIVHPNSILVVT :  74

OpSWEET    : INGIGLVVEGAYLIIFFLYSPNKKRLRMMAVLGVELVFMVAVILGVLLGAHTHEKRSMIVGILCVFFGSI : 418
OsSWEET6a : ---------------------------------------------------------------------- :   -
OsSWEET6b : INGIGLIVEGTYLFIFFLYSPNKKRLRMLAVLGVELVFMLAVILGVLLSAHTHKKRSMIVGILCVFFGSI : 144

OpSWEET    : MYFSPLTIMGKVIKTKSVEYMPFFLSLVCFLNGVCWTAYALIRFDIYVTIPNGLGALFGAIQLILYACYY : 488
OsSWEET6a : ---------------------------------------------------------------------- :   -
OsSWEET6b : MYFSPLTIMGKVIKTKSVEYMPFFLSLVCFLNGVCWTAYALIRFDIYVTIPNGLGAIFGAIQLILYACYY : 214

OpSWEET    : RTTPKKTKASKDVEMPSVVVSGPGAATAAAAA---SVTVER : 526
OsSWEET6a : ----------------------------------------- :   -
OsSWEET6b : RTTPKKTKAAKDVEMPSVI-SGPGAAATASGGSVVSVTVER : 254
```

Figure S5. Sequence alignment of a 15-TMH SWEET from *Oryza punctata* (OpSWEET, A0A0E0JKY0) with OsSWEET6a (Q8LR09) and OsSWEET6b (Q8W0K2).

Supplemental Fig. 6

```
H3GD93 : MVDNAVFWVIKILAALTSLGMICSPALSTARIHNKRDVGVASVIPLASLLANAHNWVLYGYMVKNWFPIFWVFVFGDLAASSYIAVYWRHTTERRYVMRVLA :  102
W2WEC6 : MVDSTVFWIIKILAALTSLGMICSPALSIFRIYKKSDVGVASVIPLASLLANTHNWVLYGYMVKNWFPIFWVFVFGDIAALSYMTVYWWYTPERHYVLRVLA :  102
W2WDZ7 : ------------------------------------------------------------------------------------------------------ :    -
W2WFE5 : ------------------------------------------------------------------------------------------------------ :    -
G4Z0X0 : ------------------------------------------------------------------------------------------------------ :    -

H3GD93 : VVAAFLLATTTYAVVGGLGYLGQSRGQVGSTLGIICDVVAVCLYGAPMEKLLYVLKYKSAAFINVHMVIAGLCNNVMWIIYGAVTDNWYITSPNLLHITVNS :  204
W2WEC6 : VVTAFLLVVSTYAVVGGLGYLNQTREQVGSTLGIICDVVAVCLYGAPMEKLFHVLKYKSAVFINVHMVIAGLSNNVMWIVYGIVTDNWYIISPNMFHITVNS :  204
W2WDZ7 : ------------------------------------------------------------------------------------------------------ :    -
W2WFE5 : ------------------------------------------------------------------------------------------------------ :    -
G4Z0X0 : ------------------------------------------------------------------------------------------------------ :    -

H3GD93 : STLVLYLVFNPKTHPLPESFHTGSADEGAVSIEITPKASLSRKP--------------AMVSSSVLWAIKVLAATTSLMMICSPSISVYRIHKKQDVGVASV :  292
W2WEC6 : STLVLYLAFNPKTHPLPDSYFTTT-DDAVISIEITPKASFNRKTSGDLPSPAYQAMPLR------------------------------------------- :  262
W2WDZ7 : -----------------------------------------------------------MVASSVLWAIKVLAAMTSLMMICSPSISVYRIHKKKDVGVASV :   43
W2WFE5 : ------------------------------------------------------------------------------------------------------ :    -
G4Z0X0 : ------------------------------------------------------------------------------------------------------ :    -

H3GD93 : IPLASLLANGHMWMLYGYLVENWFPIFWIFVVGDVAALSFLAIYWRYTTERRYVGRVLATVLSVLTVVSIYAIVGGLGYTGQSRHQVGSALGFICDVVAVCL :  394
W2WEC6 : ------------------------------------------------------------------------------------------------------ :    -
W2WDZ7 : IPLASLLANGHMWMLYGYMVENWFPIFWIFVFGDIAALTFLVVYWRYTTERRYVGRVLAAVFSILAIATVYAVVGGFGHTGQSRHQVGTTLGFICDVVAVCL :  145
W2WFE5 : ------------------------------------------------------------------------------------------------------ :    -
G4Z0X0 : ------------------------------------------------------------------------------------------------------ :    -

H3GD93 : YGAPMEKLFHVLKYKSAVFINVHMVIAGLSNNCTWITYGILSSNWFIISPNILFISLNSFTLVLYTVFNPKTHPLPDNFHHD----AAESVISIELTPKGSF :  492
W2WEC6 : ------------------------------------------------------------------------------------------------------ :    -
W2WDZ7 : YGAPMEKLFHVLKYRSAVFINVHMVIAGLSNNCTWITYGILSTNWFIISPNILFISLNSFTLVLYMVFNPKTHPLPDNFHRATGPECAESVVSIELTPKESF :  247
W2WFE5 : ------------------------------------------------------------------------------------------------------ :    -
G4Z0X0 : ------------------------------------------------------------------------------------------------------ :    -

H3GD93 : SRKVGSDLPSPAAFEAMSSPLETLPCVGIMVSSASMRVVEVLSALTSLALICSPAIATARIFRSKRVGVASVIPLATLLANAHMWMLYGYMIENWFPVFWVF :  594
W2WEC6 : ------------------------------------------------------------------------------------------------------ :    -
W2WDZ7 : NRKVNNELPSP-AFEAMQSPLETLPW---------------------------------------------------------------------------- :  272
W2WFE5 : -----------------------------MVSSAAMRVVEIFSALTSLSLICSPAIAMSHIFSQKNVGVASVFPLATLLANSHMWMLYGYMIKNWFPVFWVF :   73
G4Z0X0 : ------------------------------------------------------------------------------------------------------ :    -
                                                                                                        6
H3GD93 : LFGDAAGLSYLAVYWRFTPERRQVARVLAVTFVVLFVATLYAVVGGFGYTGQTRGQVGSTVGVLCDIVAVCLYGAPMEKLFQVLKYRSAAFINAHMVVASLA :  696
W2WEC6 : ------------------------------------------------------------------------------------------------------ :    -
W2WDZ7 : ------------------------------------------------------------------------------------------------------ :    -
W2WFE5 : LFGDAVGVLYLVVYWRYTPDRRYVARVLGVTLVVLFAATLYAIIGGFGYTGQTRGQIGSTVGVLCDVVAVCLYGAPMEKLFQVLKYRSAAFINVHMVIASLS :  175
G4Z0X0 : ------------------------------------------------------------------------------------------------------ :    -

H3GD93 : NNVMWFTYGMLTDNWYIISPNIVFVTLNSSTLVLYLVFYPKSHPLPADFNQSTAEDSPK----TSVSCKAGVDAPSPAFEALQSPPETLHCSPLRSGCLAVH :  794
W2WEC6 : ------------------------------------------------------------------------------------------------------ :    -
W2WDZ7 : ------------------------------------------------------------------------------------------------------ :    -
W2WFE5 : NNIMWFTYGVLTENWYIISPNILFITLNSSTLVLYLVFNPKTHPLPDDFNLHATIQDSVIESSNKTVYSEGVNSCSPAFDAVRSPPGTL------------- :  264
G4Z0X0 : ------------------------------------------------------------------------------------------------------ :    -

H3GD93 : PRGHVEVDEGVSRVGDRHFDFCYVPTMGVWLVVLKAATSIAAMTMCLSPIPSIHRIYRTKDTGEVAVLPLVALWISCHLWMIYGYVTNDVFPLLVTYLVGEV :  896
W2WEC6 : ------------------------------------------------------------------------------------------------------ :    -
W2WDZ7 : ------------------------------------------------------------------------------------------------------ :    -
W2WFE5 : ----------------------------------------------V------------------------------------------------------- :  265
G4Z0X0 : -----------------------------------------------SAITSVYRVYTNKDTGEVAVLPLVALWISCHLWMIYGYVTNDIFPLLVTYLVGEV :   55

H3GD93 : LAACYVGVYFRYTKHRSYTLKAVAFALAFTAIGTTYAVLGREGVTDQSLSTVGNIMGWITAGGSFLLYTSPFETIKRVLRTKSGASIPIALCCAGFVSNALW :  998
W2WEC6 : ------------------------------------------------------------------------------------------------------ :    -
W2WDZ7 : ------------------------------------------------------------------------------------------------------ :    -
W2WFE5 : ------------------------------------------------------------------------------------------------------ :    -
G4Z0X0 : LAACYVAVHFCYTKHRAYTIKAVAFALTFTALGTTYAVLGREGVTYQSLSAVGNVMDWITAGGSFLLYTSPFETIKRVLQTKSGASIPIALCCAGLVSNSLW :  157

H3GD93 : ILYGLVESDMFVFGLGIFCTTMPLVQITLYFLFTPKQQQSCSPDAVDASKDHNTPESRELAEIVVTP--SP-GAHAAFSSDAANFHAAPSPVLA : 1089
W2WEC6 : ---------------------------------------------------------------------------------------------- :    -
W2WDZ7 : ---------------------------------------------------------------------------------------------- :    -
W2WFE5 : ---------------------------------------------------------------------------------------------- :    -
G4Z0X0 : VLYGLVVSDMFVFGLGVFCTTLPLIQIILYLVFNPNRNQAFGVESSET---------KELTDMIASTSIDPGASTLEFASSVADFHAAPSPA-- :  240
```

Figure S6. Sequence alignment of the superSWEET (H3GD93) with four SWEETs (indicated by their Uniprot IDs) from *Phytophthora*.
